# Supplementary material for: A Comprehensive Analysis of Programmed Cell Death-Associated Genes for Tumor Microenvironment Evaluation Promotes Precise Immunotherapy in Patients with Lung Adenocarcinoma
Source: J Pers Med. 2023 Mar 6;13(3):476. doi: 10.3390/jpm13030476 (PMC10058589; doi:10.3390/jpm13030476)

Supplementary Figures

Supplementary Figure S1. PCA analysis for each dataset.

Supplementary Figure S2. Identification of apoptosis-, ferroptosis-, and autophagy-related differentially expressed genes.

Supplementary Figure S3. Pathway enrichments of apoptosis-, ferroptosis-, and autophagy-related differentially expressed genes.

Supplementary Figure S4. LASSO regression analysis of apoptosis-related prognostic genes.

Supplementary Figure S5 Prognostic analysis of apoptosis-related risk score in the training and validation cohorts.

Supplementary Figure S6 LASSO regression analysis of ferroptosis-related prognostic genes.

Supplementary Figure S7 Prognostic analysis of ferroptosis-related risk score in the training and validation cohorts.

Supplementary Figure S8 LASSO regression analysis of autophagy-related prognostic genes.

Supplementary Figure S9 Prognostic analysis of autophagy-related risk score in the training and validation cohorts.

Supplementary Figure S10 Co-expression network for programmed death genes.

Supplementary Figure S11 Pathway enrichments of programmed cell death risk score model.

Supplementary Figure S12 The differential expressed genes regrard as the independent factors for the prognosis of LUAD.

Supplementary Figure S1 PCA analysis for each dataset.

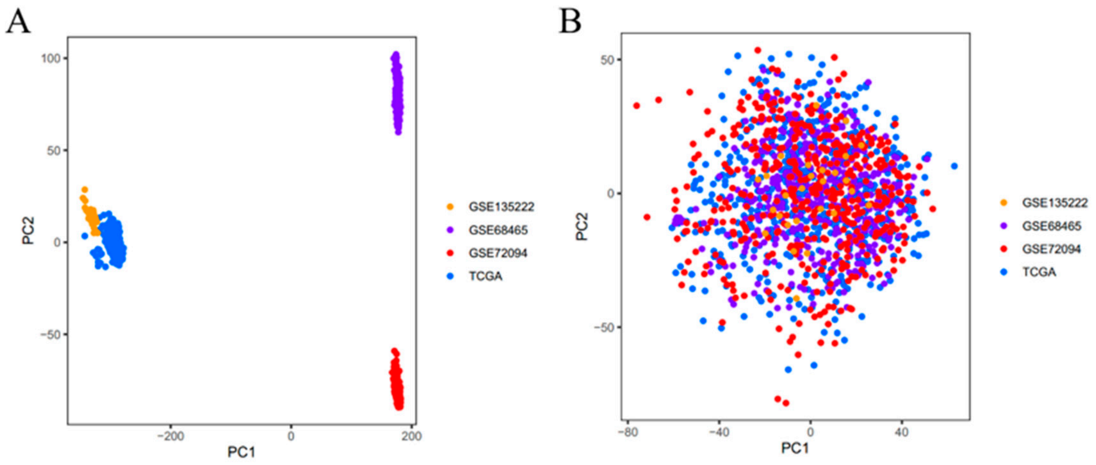

(A) datasets before batch correction. (B)datasets after batch correction.

Supplementary Figure S2. Identification of apoptosis-, ferroptosis-, and autophagy-related differentially expressed genes.

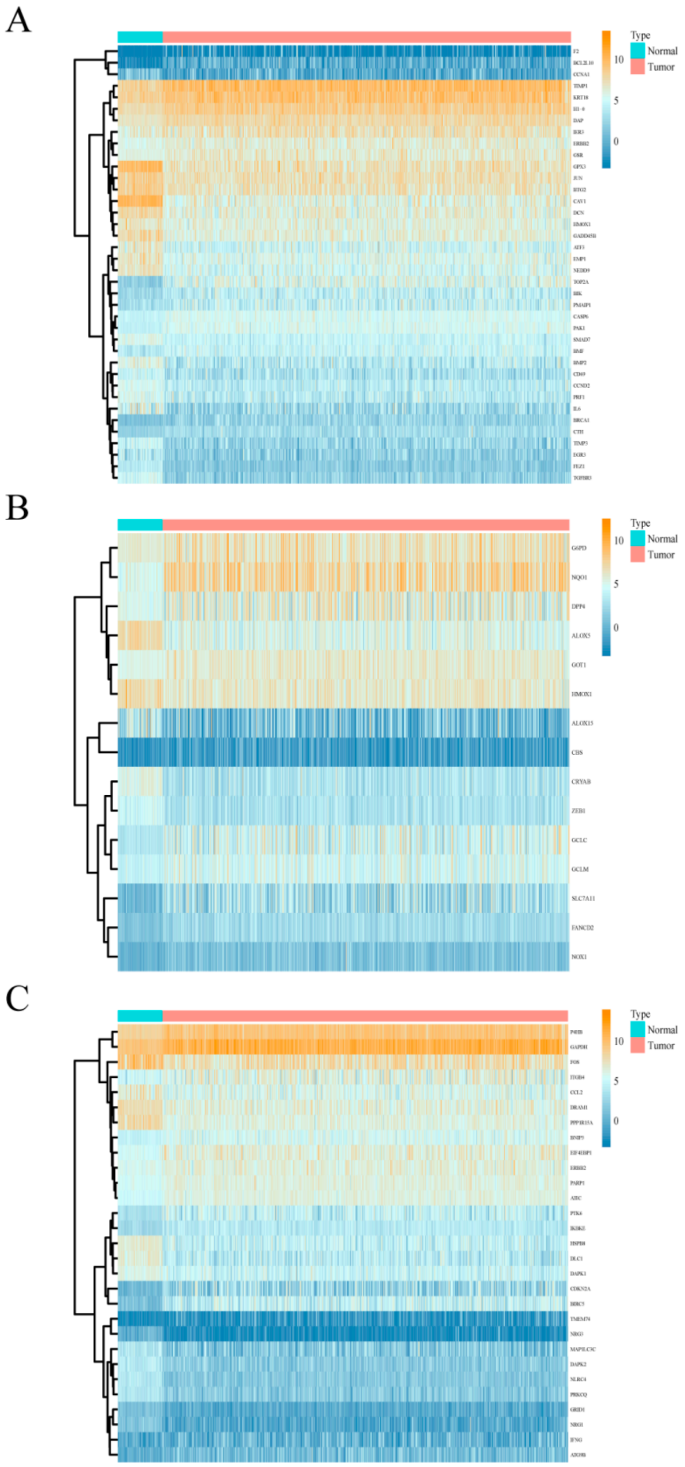

(A) Heatmaps of differentially expressed apoptosis-related genes. (B) Heatmaps of differentially expressed ferroptosis-related genes. (C) Heatmaps of differentially expressed autophagy-related genes.

Supplementary Figure S3. Pathway enrichments of apoptosis-, ferroptosis-, and autophagy-related differentially expressed genes.

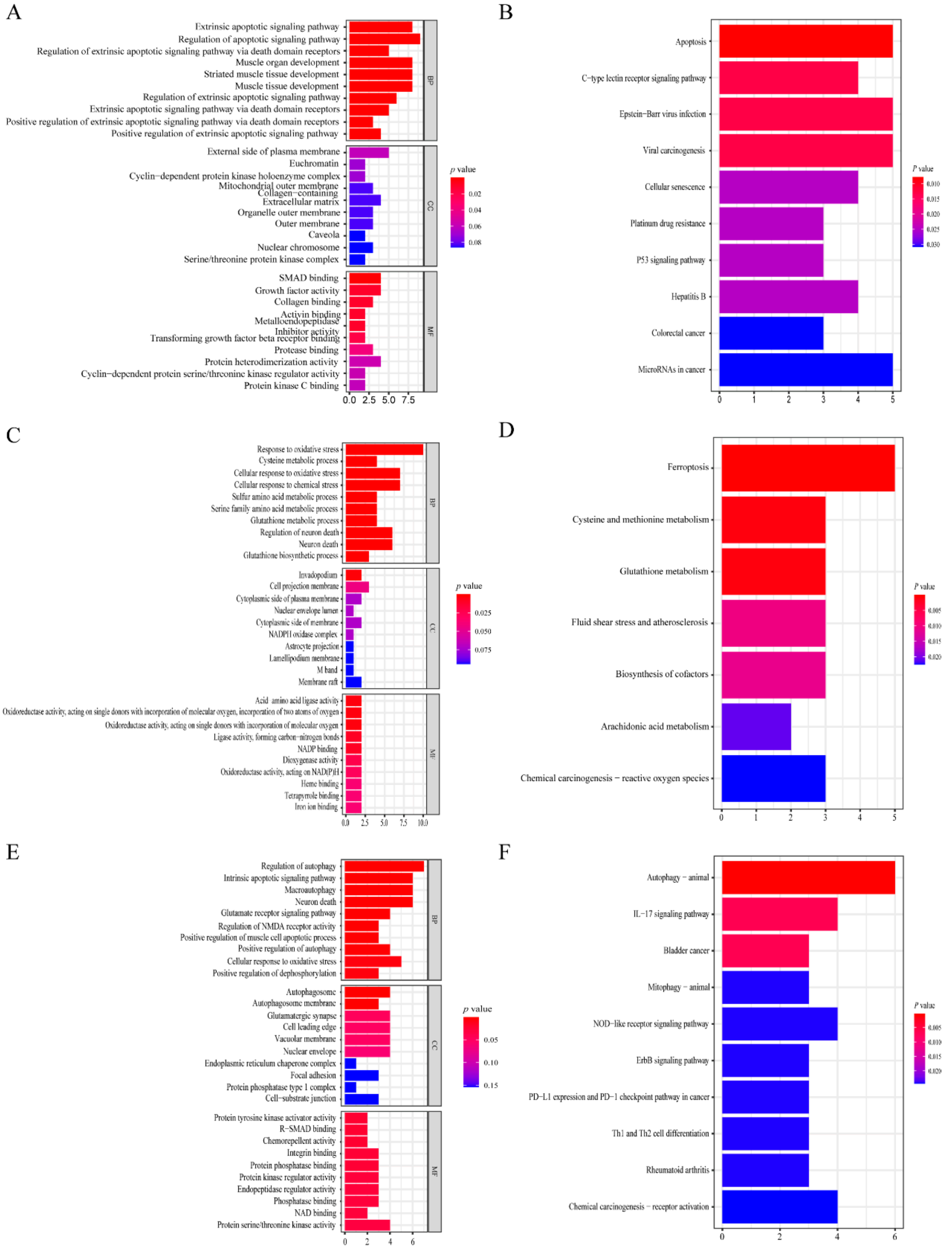

(A) GO enrichment of differentially expressed apoptosis-related genes. (B) KEGG enrichment of differentially expressed apoptosis-related genes. (C) GO enrichment of differentially expressed ferroptosis-related genes. (D) KEGG enrichment of

differentially expressed ferroptosis-related genes. (E) GO enrichment of differentially expressed autophagy-related genes. (F) KEGG enrichment of differentially expressed autophagy-related genes.

Supplementary Figure S4. LASSO regression analysis of apoptosis-related prognostic genes.

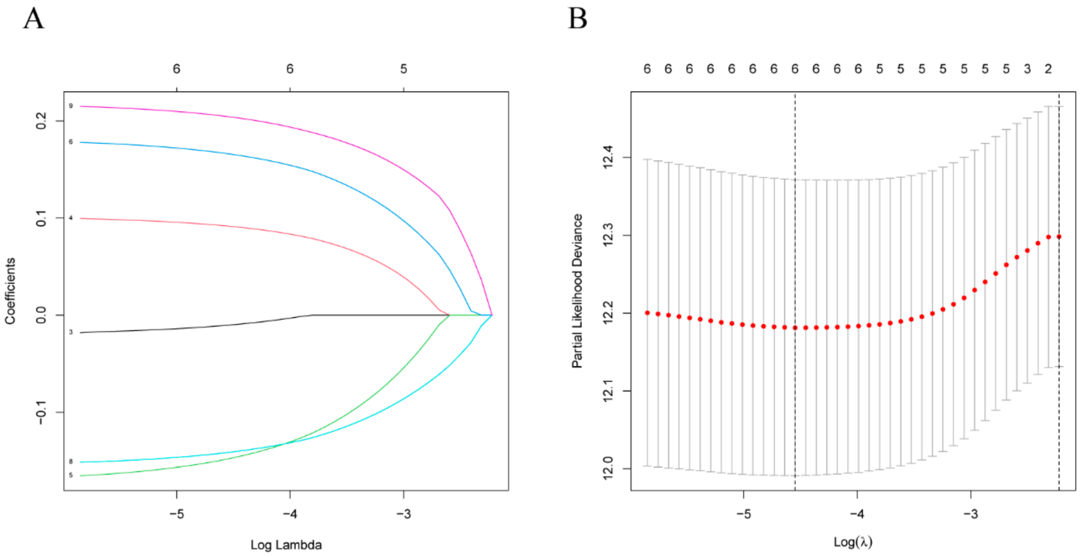

(A)(B) LASSO coefficient profile plots of the apoptosis-related prognostic genes showing that the variations in the size of the coefficients of parameters shrink with an increasing value of the k penalty.

Supplementary Figure S5. Prognostic analysis of apoptosis-related score in the training and validation cohorts.

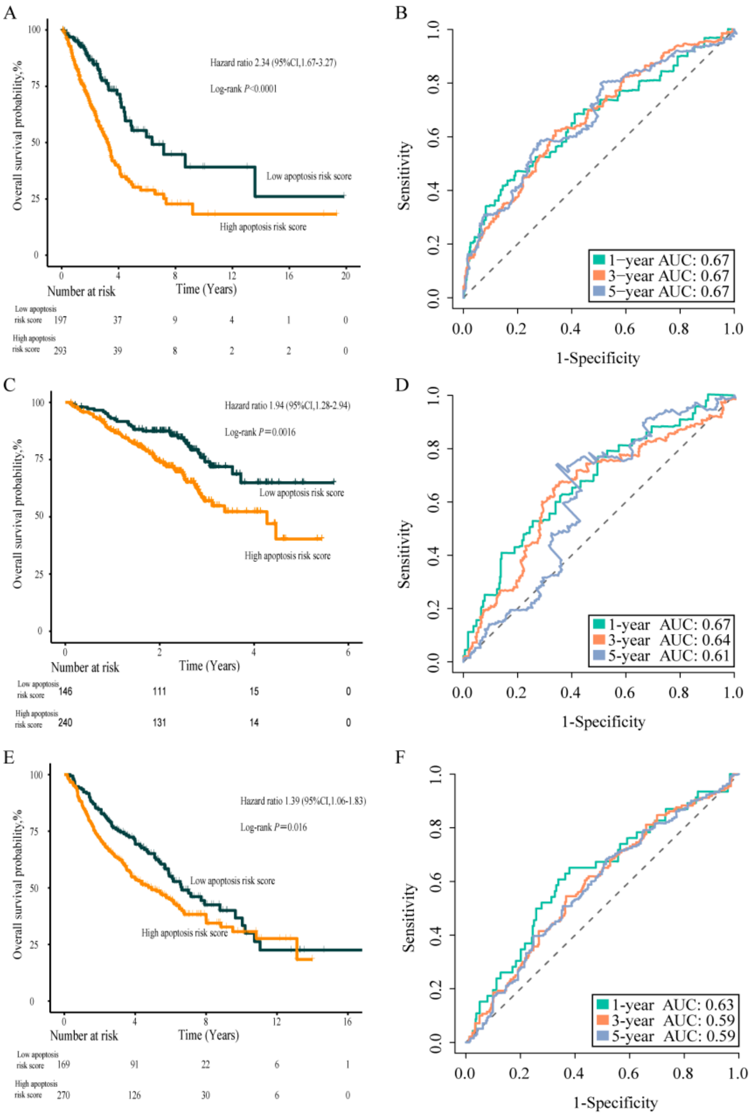

(A) Kaplan-Meier survival curve of the patients from the training cohort assigned to different groups based on their apoptosis risk score. (B) Time-related ROC analysis showing the prognostic performance of the apoptosis risk score formula in the training cohort. (C) Kaplan-Meier survival curve of the patients from validation cohort 1 distributed to different groups based on their apoptosis risk score. (D) Time-related ROC analysis showing the prognostic performance of the apoptosis score formula in validation cohort 1. (E) Kaplan-Meier survival curve of the patients from the validation cohort 2 distributed in different groups based on their apoptosis risk score. (F) Time-related ROC analysis showing the prognostic performance of the apoptosis risk score formula in validation cohort 2.

Supplementary Figure S6. LASSO regression analysis of ferroptosis-related prognostic genes.

A B

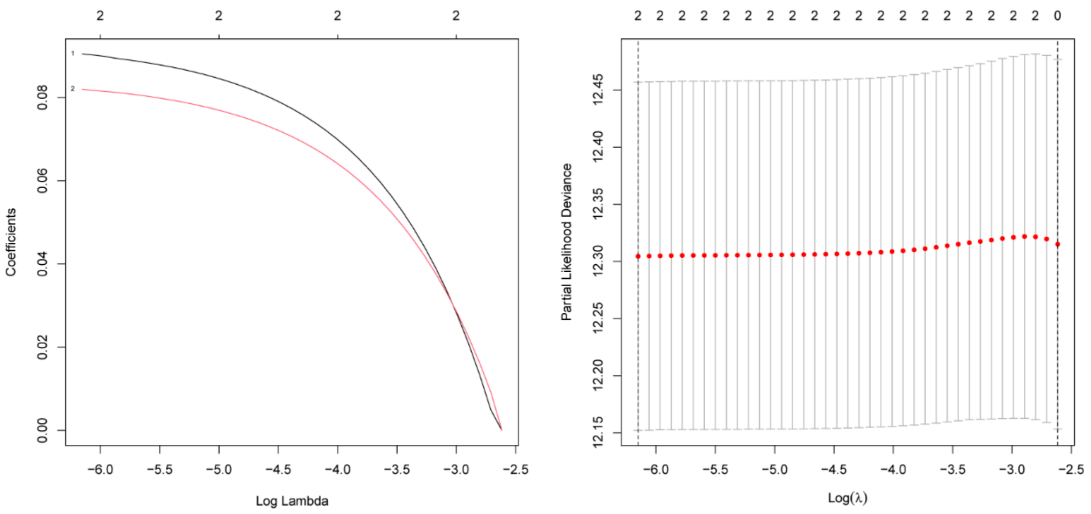

(A) (B) LASSO coefficient profile plots of the ferroptosis-related prognostic genes showing that the variations in the size of the coefficients of parameters shrink with an increasing value of the k penalty.

Supplementary Figure S7. Prognostic analysis of ferroptosis-related score in the training and validation cohorts.

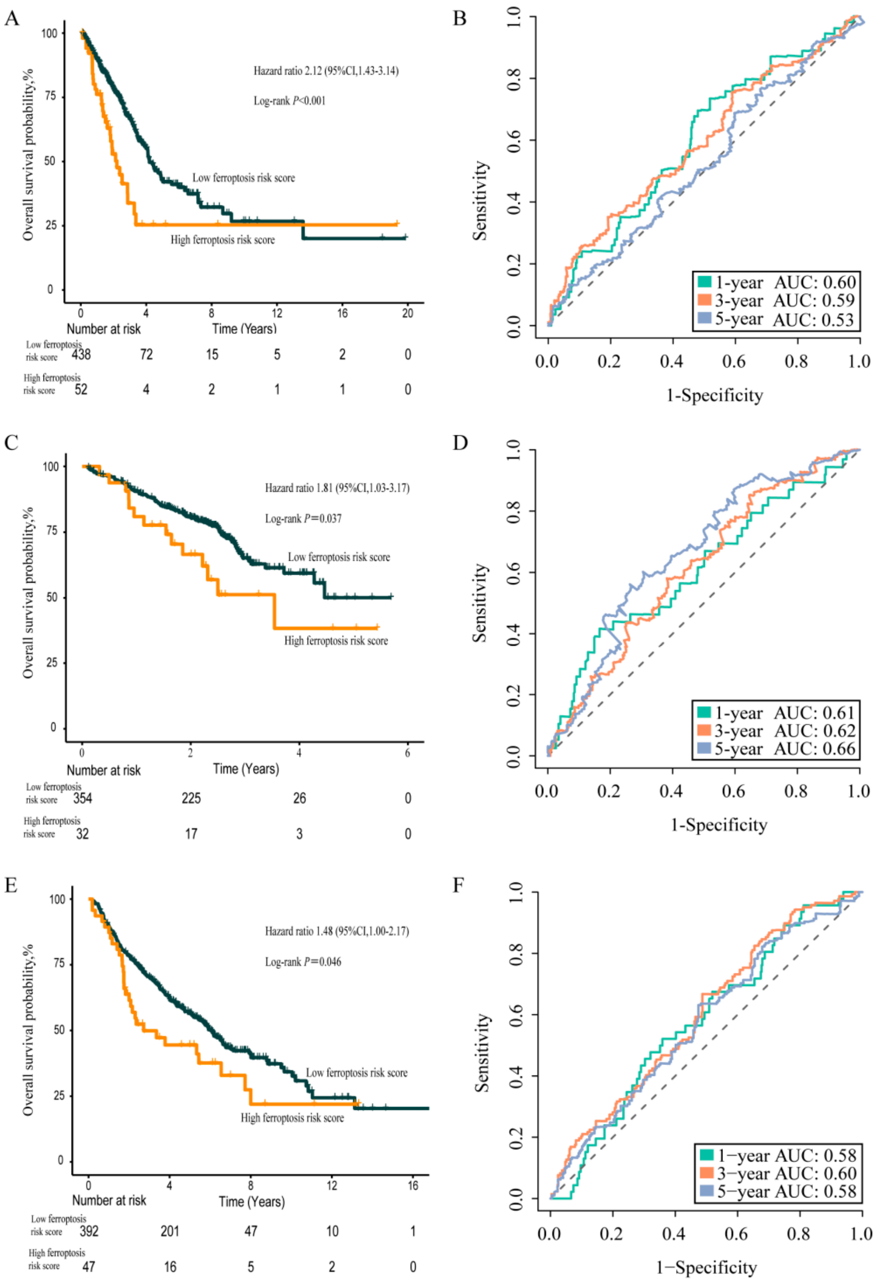

(A) Kaplan-Meier survival curve of the patients from the training cohort assigned to different groups based on their ferroptosis risk score. (B) Time-related ROC analysis showing the prognostic performance of the ferroptosis risk score formula in the training cohort. (C) Kaplan-Meier survival curve of the patients from validation cohort 1 distributed in the different groups based on their ferroptosis risk score. (D) Time-related ROC analysis showing the prognostic performance of the ferroptosis risk score formula in validation cohort 1. (E) Kaplan-Meier survival curve of the patients from the validation cohort 2 distributed in different groups based on their ferroptosis risk score.

(F) Time-related ROC analysis showing the prognostic performance of the ferroptosis risk score formula in validation cohort 2.

Supplementary Figure S8. LASSO regression analysis of autophagy-related prognostic genes.

A B

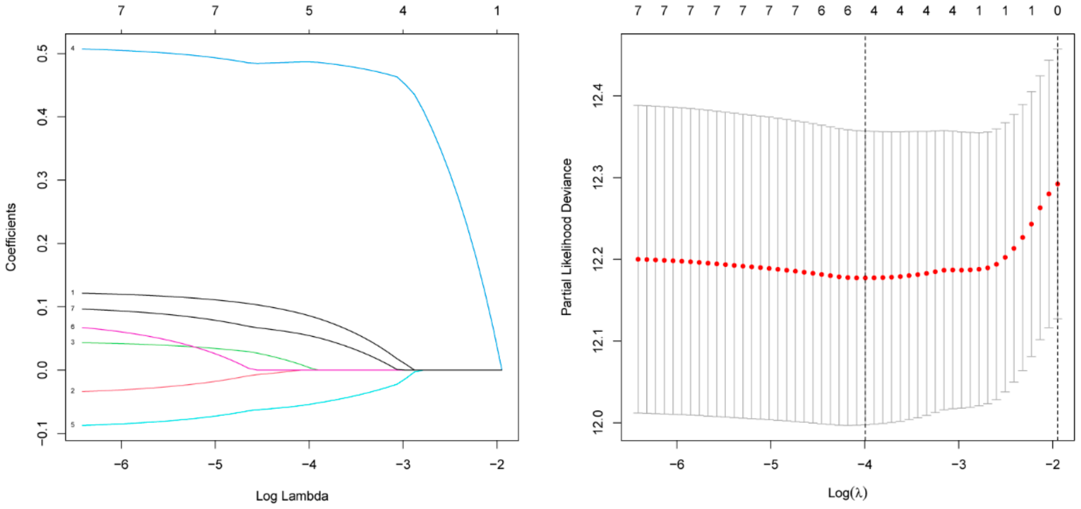

(A) (B) LASSO coefficient profile plots of the autophagy-related prognostic genes showing that the variations in the size of the coefficients of parameters shrink with an increasing value of the k penalty.

Supplementary Figure S9. Prognostic analysis of autophagy-related score in the training and validation cohorts.

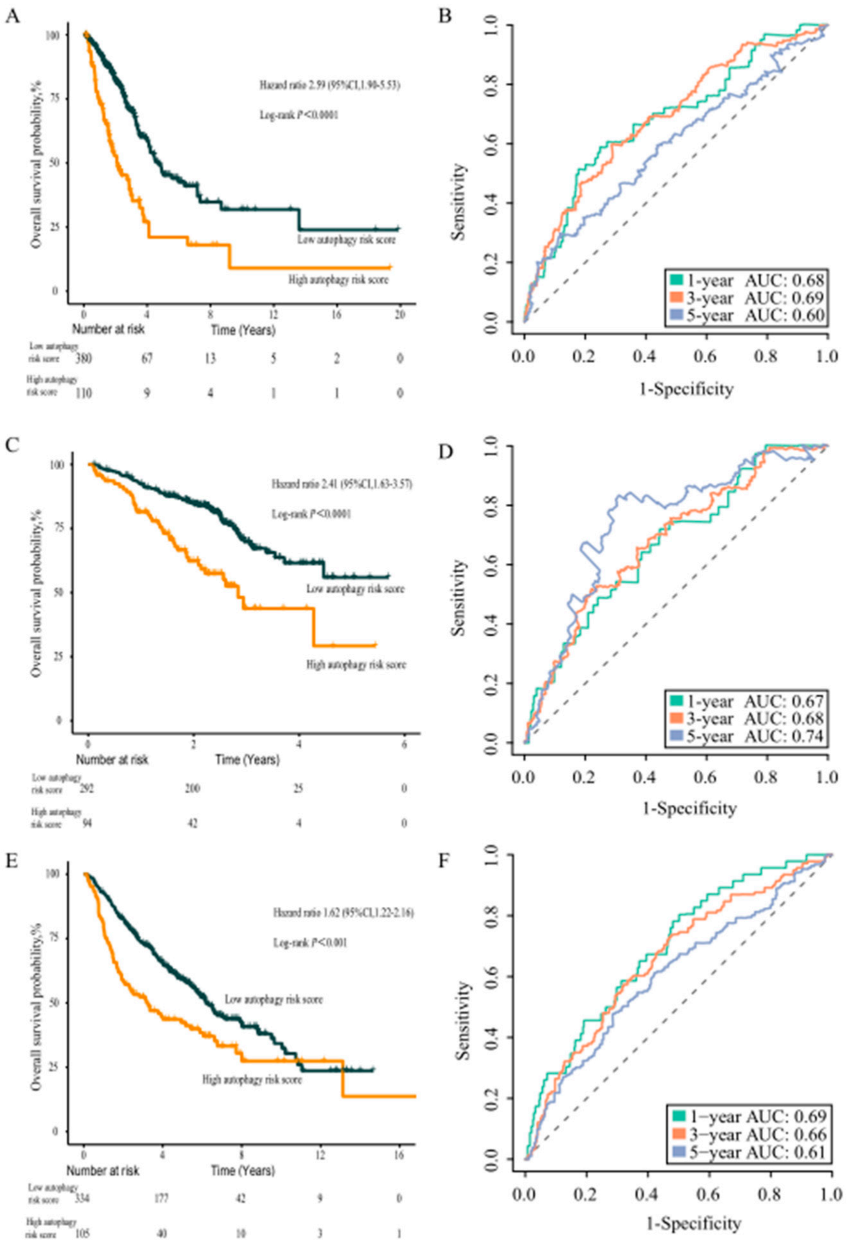

(A) Kaplan-Meier survival curve of the patients from the training cohort distributed in different groups based on their autophagy risk score. (B) Time-related ROC analysis showing the prognostic performance of the autophagy risk score formula in the training cohort. (C) Kaplan-Meier survival curve of the patients from validation cohort 1 distributed in different groups based on to their autophagy risk score. (D) Time-related ROC analysis showing the prognostic performance of the autophagy risk score formula in the validation cohort 1. (E) Kaplan-Meier survival curve of the patients from validation cohort 2 distributed in different groups based on their autophagy risk score.

(F) Time-related ROC analysis showing the prognostic performance of the autophagy risk score formula in validation cohort 2.

Supplementary Figure S10 Co-expression network for programmed death genes.

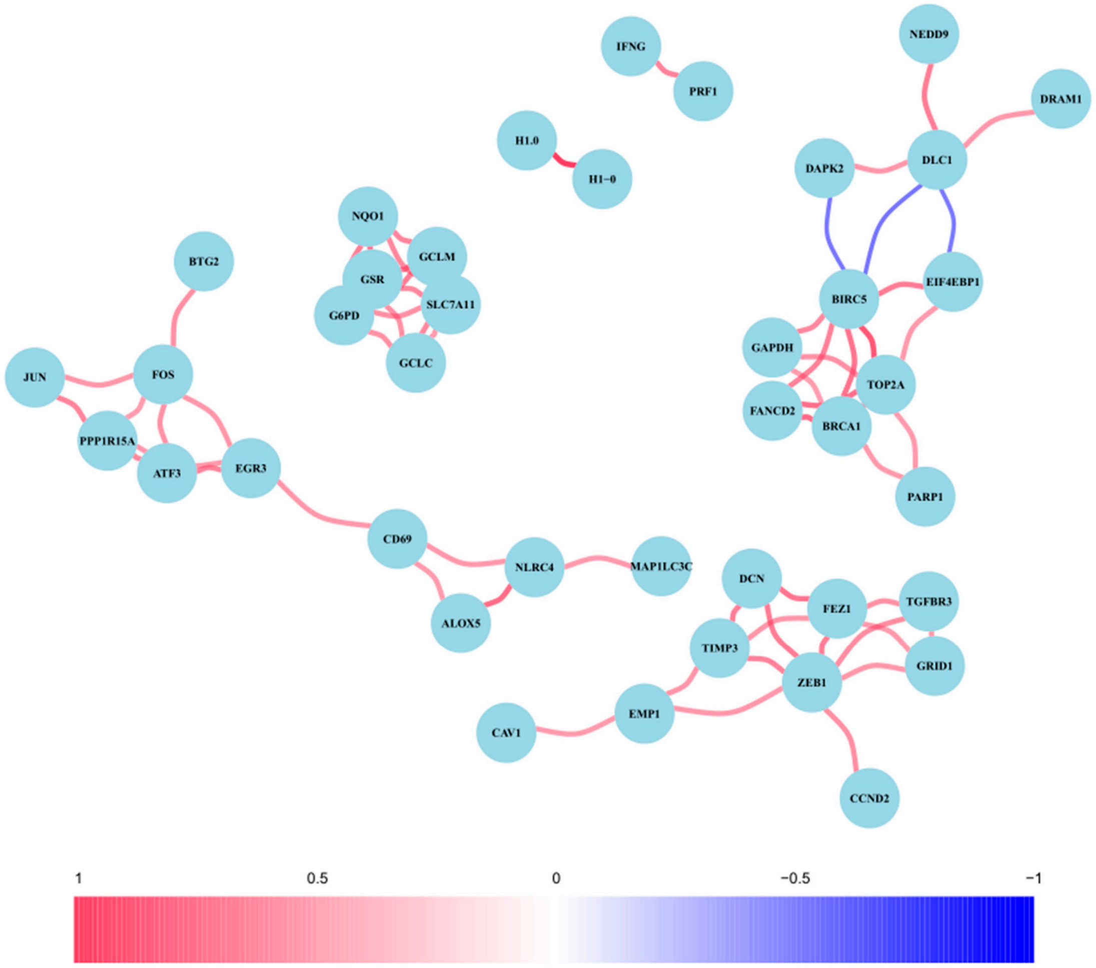

Supplementary Figure S11. Pathway enrichments of programmed cell death risk score model.

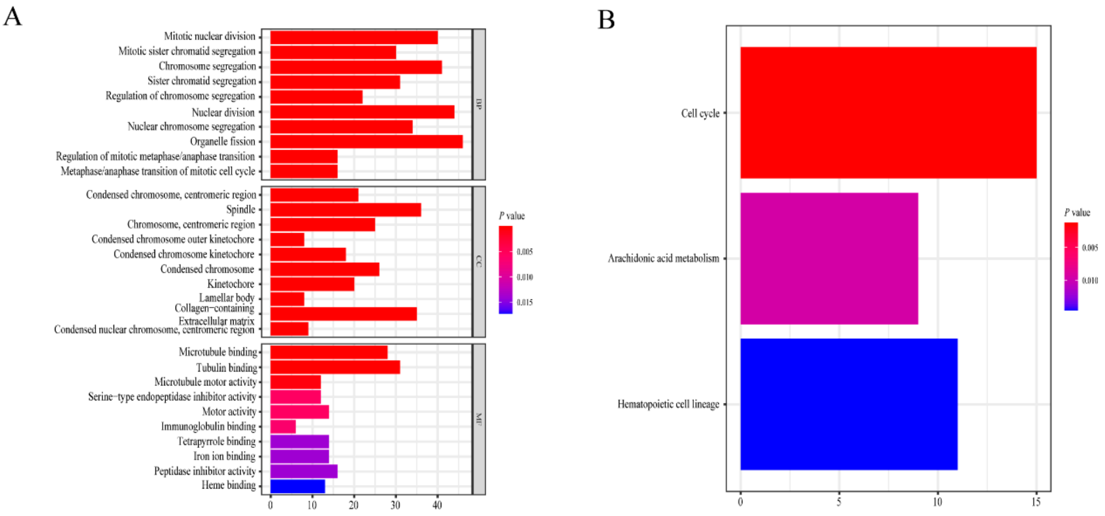

(A) Comparison of the GO enrichment between the high and low programmed cell death risk score groups from the training cohort. (B) Comparison of the KEGG pathways between the high and low programmed cell death risk score groups from the training cohort.

Supplementary Figure S12 The differential expressed genes regrard as the independent factors for the prognosis of LUAD.

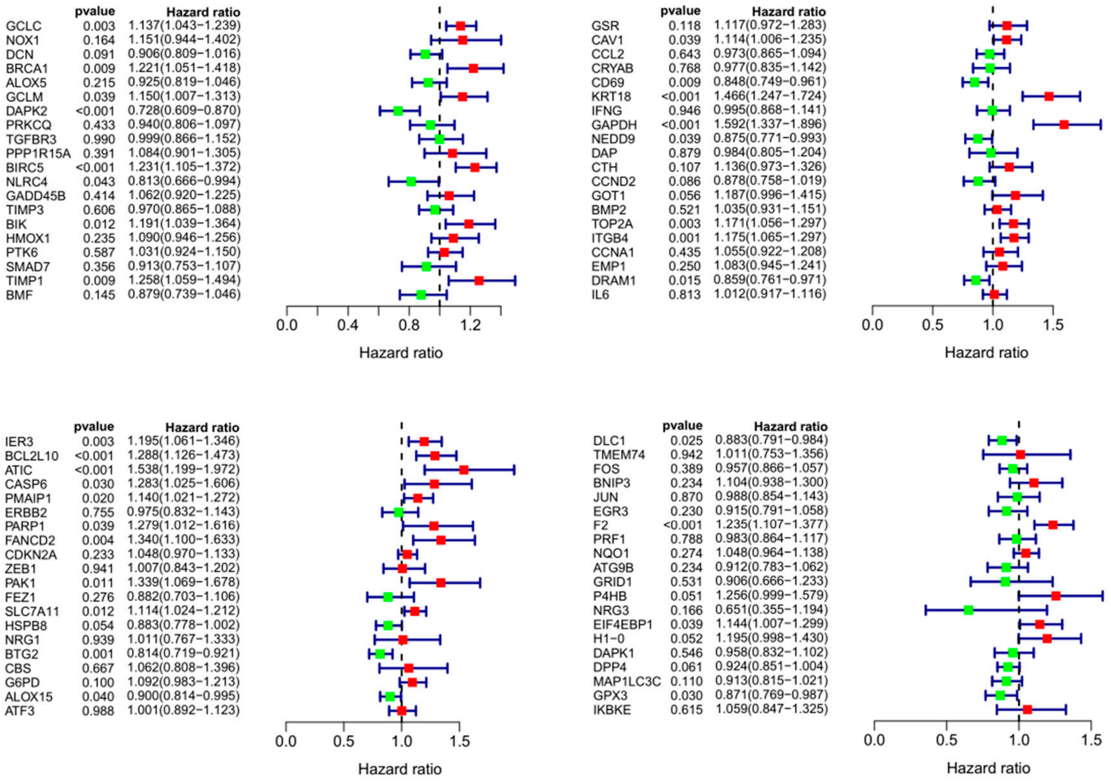

Supplement: Supplementary file 1 [file jpm-13-00476-s001.zip › Supplementary Figures.pdf]
