# Supplementary material for: A Comprehensive Analysis of Programmed Cell Death-Associated Genes for Tumor Microenvironment Evaluation Promotes Precise Immunotherapy in Patients with Lung Adenocarcinoma
Source: J Pers Med. 2023 Mar 6;13(3):476. doi: 10.3390/jpm13030476 (PMC10058589; doi:10.3390/jpm13030476)
Supplement: Supplementary file 1 [file jpm-13-00476-s001.zip › Supplementary Tables.pdf]

Supplementary tables

Supplementary Table S1. Summary of primers of each gene.

Supplementary Table S2. Clinical information of patients with LUAD in this study.

Supplementary Table S3. Summary of apoptosis-related differentially expressed genes.

Supplementary Table S4. Summary of ferroptosis-related differentially expressed genes.

Supplementary Table S5. Summary of autophagy-related differentially expressed genes.

Supplementary Table S6. Summary of the differentially expressed genes between high and low programmed cell death risk score group.

Supplementary Table S7. GO enrichment analysis of differentially expressed genes between high and low programmed cell death score group.

Supplementary Table S8. KEGG enrichment analysis of differentially expressed genes between high and low programmed cell death score group.

Supplementary Table S1. Summary of primers of each gene

Gene	Primner direction	Sequence
F2	Forward primer	5'-AGATCGGCATGTCACCTTGG-3'
	Reverse primer	5'-TGAAGTTCTTGTCCCAGGGC-3'
GPX3	Forward primer	5'-AAGAGCTTGCACCATTCGGT-3'
	Reverse primer	5'-CTCCACCTGGTCGGACATAC-3'
BCL2L10	Forward primer	5'-CCCTTTCCACTGGCTTTTTGG-3'
	Reverse primer	5'-TTAGTTGGTCACAGTTGGGCA-3'
BTG2	Forward primer	5'-CACCTGCAAGAACCAAGTGC-3'
	Reverse primer	5'-AGGTATGTGGTGGCCTGTTG-3'
KRT18	Forward primer	5'-GGCTCTGGTTCCCGGATCTC-3'
	Reverse primer	5'-GTCGTTCAGGCTTTGCATGG-3'
GCLC	Forward primer	5'-TAAGCCCCCTCCTCCAAACT-3'
	Reverse primer	5'-GGATCACTCTGGTGAGCAGT-3'
ITGB4	Forward primer	5'-GACATCCGGGCCCTAGACA-3'
	Reverse primer	5'-GTCCCATCCACGTGCTCAAG-3'
SLC7A11	Forward primer	5'-ATGCTGGCAGCCATCTCTTT-3'
	Reverse primer	5'-TAGACCAAACCCACGATGCC-3'
GAPDH	Forward primer	5'-AAATCAAGTGGGGCGATGCT-3'
	Reverse primer	5'-CAAATGAGCCCCAGCCTTCT-3'
BIRC5	Forward primer	5'-GGTGATGTGGATCTCGGCTT-3'
	Reverse primer	5'-TTTAAGGAAAGGCCGTGGCA-3'
ACTB	Forward primer	5'-TGGAACGGTGAAGGTGACAG-3'
	Reverse primer	5'-AACAACGCATCTCATATTTGGAA-3'

Supplementary Table S2. Clinical information of patients with LUAD in this study

**Cohorts**	**TCGA-LUAD**	**GSE68465**	**GSE72094**	**GSE135222**
**Number of patients**	490	439	386	27
**Age(Mean±SD)**	65±10.01	64±10.08	69±9.41	62±8.95
**Follow up time (Mean±SD)(years)**	2.54±2.46	4.41±3.01	2.24±1.05	0.35±0.40
**Follow up status**				
Alive(or Non-progress)	178(36.33%)	206(46.92%)	277(71.76%)	6(22.2%)
Dead(or Progression)	312(63.67%)	233(53.08%)	109(28.24%)	21(77.8%)
**Gender**				
Male	228(46.53%)	221(50.34%)	168(43.52%)	22(81.2%)

Female	262(53.47%)	218(49.66%)	218(56.48%)	5(18.5%)
**Tumor stage**				
Stage I	263(53.67%)	—	246(63.73%)	—
Stage II	115(23.47%)	—	65(16.84%)	—
Stage III	79(16.12%)	—	56(14.51%)	—
Stage IV	25(5.1%)	—	14(3.63%)	—
Unknown	8(1.63%)	—	5(1.3%)	—
**T stage**				
T1	163(33.27%)	—	—	—
T2	263(53.67%)	—	—	—
T3	43(8.78%)	—	—	—
T4	18(3.67%)	—	—	—
Unknown	3(0.61%)	—	—	—
**M stage**				
M0	324(66.12%)	—	—	—
M1	24(4.9%)	—	—	—
Unknown	142(28.98%)	—	—	—
**N stage**				
N0	317(64.69%)	—	—	—
N1	92(18.78%)	—	—	—
N2	68(13.88%)	—	—	—
N3	2(0.41%)	—	—	—
Unknown	11(2.24%)	—	—	—

LUAD: Lung Adenocarcinoma

Supplementary Table S3. Summary of apoptosis-related differentially expressed genes

Genes	Normal mean	Tumor mean	logFC	FDR
ERBB2	53.95151	115.5234	1.09845	8.24E-12
TOP2A	4.100736	65.2725	3.992521	1.01E-32
DCN	263.5448	98.50812	-1.41973	3.82E-22
CASP6	21.29729	42.91521	1.010819	1.13E-23
BRCA1	2.243802	6.602468	1.55706	3.15E-16
PAK1	19.37819	45.42825	1.229156	8.33E-27
GSR	62.57321	130.0547	1.055502	1.76E-07
FEZ1	16.18518	3.800841	-2.09028	1.41E-31
CCND2	43.69996	20.0543	-1.12372	9.79E-23
EMP1	187.8912	58.30875	-1.68811	3.39E-18
H1-0	194.3844	455.7345	1.229281	6.43E-21
IL6	90.02395	12.87702	-2.80551	2.40E-14
JUN	404.5419	196.337	-1.04296	9.07E-14
BMP2	74.08091	22.55298	-1.71578	2.29E-23
SMAD7	53.89011	25.83579	-1.06065	6.90E-22
NEDD9	135.5231	47.35134	-1.51706	1.94E-24
F2	0.014541	2.342485	7.331734	1.08E-08
CTH	4.491548	10.27525	1.193888	5.11E-13
GPX3	977.0999	213.3145	-2.19552	5.95E-31
BIK	6.323503	25.78731	2.027865	4.46E-20
TGFBR3	35.20263	6.301537	-2.48191	4.23E-31
PMAIP1	9.753947	32.16271	1.721331	5.06E-12
BCL2L10	0.101511	1.922282	4.24311	4.57E-21
IER3	104.2548	209.0634	1.003827	1.75E-08
CD69	28.71894	13.30273	-1.11028	2.54E-14
HMOX1	237.1725	85.0041	-1.48033	2.58E-08
DAP	122.6047	252.4932	1.04223	9.07E-22
PRF1	49.4246	20.57506	-1.26433	1.19E-16
CCNA1	4.1513	1.830098	-1.18164	7.08E-09
EGR3	19.82495	6.080861	-1.70497	1.01E-11
ATF3	147.5042	44.3293	-1.73442	1.27E-15
GADD45B	295.7206	90.89111	-1.70202	8.87E-19
CAV1	1122.195	108.187	-3.37473	1.86E-33
TIMP3	29.2944	8.180837	-1.8403	1.17E-23
BTG2	431.0148	178.6295	-1.27077	3.50E-18
TIMP1	337.2161	1000.811	1.569425	1.22E-21
BMF	14.24	28.74405	1.013314	4.60E-12
KRT18	341.9933	781.8987	1.193013	3.57E-16

Supplementary Table S4. Summary of ferroptosis-related differentially expressed genes

Genes	Normal mean	Tumor mean	logFC	FDR
GOT1	38.15677	77.73569	1.026638	5.47E-16
G6PD	61.07184	160.4975	1.393972	0.001302
GCLC	9.032639	45.41128	2.329831	5.61E-11
NQO1	36.12424	301.9941	3.063481	1.21E-21
FANCD2	2.065724	6.343254	1.618576	7.09E-28
CRYAB	43.11565	11.48605	-1.90833	5.70E-28
ALOX15	33.00706	7.94942	-2.05385	5.32E-13
DPP4	35.54338	91.14569	1.358594	0.00305
HMOX1	237.1725	85.0041	-1.48033	2.02E-08
GCLM	15.61372	33.44879	1.09914	6.63E-07
ZEB1	22.1229	9.016497	-1.2949	1.26E-22
NOX1	0.971572	2.753516	1.502881	2.31E-15
SLC7A11	1.732222	15.42726	3.154786	7.39E-17
ALOX5	148.4852	44.46523	-1.73957	5.70E-28
CBS	0.217825	0.566942	1.38003	0.001302

Supplementary Table S5. Summary of autophagy-related differentially expressed genes

Genes	Normal mean	Tumor mean	logFC	FDR
ERBB2	53.95151	115.5234	1.09845	1.02E-11
ITGB4	36.24137	117.294	1.69442	9.64E-10
PTK6	9.83288	32.77381	1.736858	1.48E-14
PARP1	44.00246	89.43756	1.023297	9.86E-22
P4HB	356.6344	790.1354	1.147654	1.11E-26
DRAM1	226.9468	112.6859	-1.01005	1.97E-19
ATIC	33.51486	81.9049	1.289149	5.71E-29
BNIP3	23.99594	54.96109	1.19562	4.69E-16
TMEM74	0.12045	0.63766	2.404354	0.000189
GAPDH	698.9194	2370.165	1.76179	3.21E-27
CDKN2A	1.612464	19.09732	3.566031	9.02E-13
NLRC4	15.4004	3.922144	-1.97325	4.52E-31
GRID1	2.475573	0.964091	-1.36052	1.90E-22
NRG3	1.304533	0.240737	-2.438	7.72E-28
NRG1	3.313373	1.234046	-1.4249	1.14E-24
DAPK2	17.94294	4.777742	-1.90902	8.40E-29
DLC1	108.9355	23.80691	-2.19402	8.42E-29
IFNG	1.069693	2.512608	1.231989	0.02111
IKBKE	7.507105	17.43193	1.215404	1.97E-23
FOS	1019.161	323.7209	-1.65456	1.02E-13
BIRC5	2.568249	35.12886	3.7738	2.09E-30
EIF4EBP1	42.02699	131.569	1.646431	1.92E-16
DAPK1	69.01759	33.90928	-1.02528	7.44E-20
PRKCQ	13.95012	4.852202	-1.52357	1.02E-26
PPP1R15A	307.433	87.01793	-1.82089	3.98E-26
MAP1LC3C	15.95419	6.511978	-1.29277	6.15E-18
ATG9B	0.98434	3.64945	1.89045	8.46E-10
CCL2	195.8406	73.54487	-1.41298	1.99E-07
HSPB8	114.4183	30.23852	-1.91986	1.75E-30

Supplementary Table S6. Summary of the differentially expressed genes between high and low programmed cell death score group

Genes	low-score Mean	high-score Mean	logFC	FDR
AC007906.2	30.12636	12.97571	-1.21521	8.93E-05
AC236972.3	2.988329	0.803269	-1.89538	4.81E-20
FIBCD1	1.653714	3.712865	1.166823	0.015642
BAIAP2L2	6.236154	12.80714	1.038219	5.19E-05
TTC29	1.714596	0.771667	-1.15182	3.69E-06
CFAP43	4.278484	2.066878	-1.04965	0.000314
AC068228.2	0.697439	1.587665	1.186767	1.48E-13
DNMT3B	3.215286	7.063384	1.135412	2.27E-12
RGS20	1.032654	2.790593	1.434215	9.31E-07
AC129926.1	0.979258	2.071938	1.08122	0.000114
ZBTB16	4.368185	1.775123	-1.29912	3.99E-16
TREML3P	1.158166	3.986908	1.783428	5.75E-06
HLF	14.9668	6.789203	-1.14045	7.05E-17
SLC25A47P1	1.689881	0.579889	-1.54307	0.009695
SHOX2	0.874055	2.612878	1.579844	3.99E-08
DEPDC1B	4.134563	9.151023	1.146198	1.43E-22
TOP2A	39.9043	85.07975	1.092272	2.51E-21
CPA6	2.575996	1.127669	-1.19179	0.015249
CDIPTOSP	1.481906	3.173093	1.098436	0.001599
DES	22.56672	9.036754	-1.32032	1.51E-12
ARTN	1.585843	4.659709	1.55499	4.73E-11
PRC1	12.53121	26.58317	1.084987	6.71E-27
MGAT5B	0.505842	1.686995	1.737696	0.000392
CFAP65	1.721047	0.724446	-1.24834	3.64E-05
PLA2G12B	14.32034	5.634392	-1.34573	1.16E-11
ITGB4	72.41687	151.7571	1.067366	7.27E-12
GSTM5	2.27234	1.134189	-1.00252	1.27E-13
SFTA1P	122.4424	45.66703	-1.42288	1.00E-16
H19	76.39964	200.4182	1.391376	0.004911
AC022497.1	2.616323	1.232791	-1.08561	1.55E-16
SPAG8	3.329638	1.635674	-1.02548	1.41E-10
GAS2L2	4.079846	1.846779	-1.1435	1.02E-06
ORM2	35.10887	15.26698	-1.20142	3.12E-06
FXYD1	1.585334	0.723727	-1.13127	3.14E-18
MYBL2	35.43737	87.06983	1.296901	1.41E-23
TK1	61.72472	144.8021	1.230162	3.90E-30
UCK2	8.552243	19.39536	1.181337	3.64E-23

CRYBG2	2.608949	6.251079	1.260637	8.26E-11
RN7SL648P	3.489332	0.672245	-2.37589	0.000606
IGKV1OR9-2	2.15733	1.043328	-1.04805	0.000243
CCNE1	7.248255	16.25769	1.165416	2.16E-20
SH3PXD2A-AS1	0.79989	2.086548	1.383245	8.63E-07
APCDD1L	1.066277	2.751211	1.367485	1.55E-09
MIR23A	5.074661	2.395571	-1.08294	1.26E-05
HSPE1P18	1.915817	0.911041	-1.07237	9.49E-10
RNU5F-1	4.075511	1.950715	-1.06298	0.042897
CDA	27.90977	79.07832	1.502512	1.52E-05
TEKT1	5.897679	2.388904	-1.3038	4.41E-06
PCLAF	9.891808	19.98711	1.014764	3.39E-22
CGB5	0.543925	9.208571	4.081498	0.028367
FOSB	86.79436	35.10427	-1.30595	7.26E-07
IGF2BP2	11.06882	26.68657	1.269613	3.75E-14
LINC02253	1.047977	2.753285	1.393547	0.001521
CFAP57	3.718481	1.822314	-1.02894	1.93E-08
CCDC17	8.196631	4.005914	-1.0329	0.010444
AL669830.1	1.91248	0.804471	-1.24933	5.52E-07
TCF21	7.310376	2.58669	-1.49884	1.48E-17
TROAP	6.73147	16.15115	1.262644	4.65E-25
EDNRB	14.63079	7.073188	-1.04858	2.52E-15
ARHGAP11A	6.059427	12.28199	1.019291	2.48E-21
FLNC	6.422032	13.97251	1.12149	0.000935
APOBEC4	1.738179	0.762769	-1.18826	8.11E-06
MIR3677	7.390399	3.663046	-1.01261	1.55E-07
PSCA	34.69956	119.5963	1.785184	0.000284
TRIM15	0.882881	3.281302	1.893977	2.76E-06
RETN	17.61821	8.439004	-1.06192	0.003059
CHIAP2	2.889467	0.943572	-1.6146	1.14E-13
MPHOSPH6P1	1.670062	0.48261	-1.79097	7.14E-05
RPL26P30	4.600689	2.045636	-1.1693	3.03E-12
CYP4F3	2.650463	11.07885	2.063492	0.020737
IGHV3OR16-6	6.255745	2.710218	-1.20677	1.69E-05
PTPRN	0.715808	3.622954	2.339522	1.80E-12
PHACTR1	6.147647	2.856115	-1.10598	2.72E-23
CRHR2	1.724196	0.440842	-1.96759	6.20E-05
TPPP3	80.77486	38.25354	-1.07831	2.67E-09
S100A8	76.46546	287.3451	1.909904	0.024047
POPDC3	1.798022	5.6012	1.639325	0.000633
TNFSF11	1.986297	4.07951	1.038314	0.01761
IGHV4OR15-8	1.998383	0.996021	-1.00459	0.000229
LINC01765	2.626285	1.168136	-1.16881	2.39E-06

TNFRSF13B	2.163363	0.840929	-1.36322	1.43E-13
KIF18B	5.314455	13.28243	1.321525	3.60E-26
AC103702.2	1.80325	3.79161	1.072211	0.004318
SCN4B	5.236412	2.178629	-1.26516	1.60E-17
MIR4538	6.88791	3.361993	-1.03475	1.70E-05
CSAG1	5.744902	17.39133	1.598014	0.008919
C1orf194	9.692791	4.826487	-1.00594	0.000116
IGKV6-21	88.75929	38.32328	-1.21168	9.09E-07
SKA1	3.91702	9.176958	1.228259	7.91E-26
IGHV2-70D	88.58826	38.52276	-1.2014	0.024389
IGKV1OR1-1	1.772891	0.625026	-1.50412	0.00127
C9orf24	12.8718	5.261546	-1.29065	2.89E-06
PI16	2.346319	0.926574	-1.34042	1.82E-08
ANKRD29	12.53812	5.649658	-1.15009	3.80E-17
HIGD1B	13.79066	6.859333	-1.00755	3.43E-15
ROBO2	2.300689	0.954542	-1.26919	1.51E-13
KRT16	7.760017	32.18923	2.052446	2.56E-12
SLC14A2	6.669537	2.393531	-1.47845	2.48E-13
FCER2	2.601987	0.82178	-1.66279	2.75E-11
NKAIN4	1.13353	2.465497	1.121056	0.000378
AFP	0.107698	8.819827	6.355688	0.012536
AHNAK2	11.64323	25.62108	1.13784	3.04E-11
TTK	4.502476	10.62881	1.239189	2.69E-23
AC246787.2	1.467835	0.686687	-1.09597	1.27E-06
IGFBP1	0.791899	13.60015	4.102162	0.003263
PADI1	1.078794	5.444916	2.335491	1.75E-09
AQP4	66.74322	29.27621	-1.18889	2.10E-11
IFNWP19	0.942558	2.471857	1.390943	1.80E-05
CDKN3	10.04808	22.05759	1.134355	3.45E-22
HTR3A	6.061837	14.48517	1.25675	0.007187
ITLN1	167.7119	3.54727	-5.56313	4.98E-05
STAC2	3.81538	1.40893	-1.43723	0.017318
EFCAB1	3.525138	1.610729	-1.12997	4.41E-06
CASC19	0.844756	2.410954	1.512998	3.50E-05
TRNP1	27.36431	58.57509	1.097992	4.45E-05
NKAIN1	0.612337	1.947271	1.669055	9.27E-09
PIH1D3	1.579204	0.663485	-1.25106	4.31E-06
AC027277.2	1.872859	0.91098	-1.03975	2.20E-08
PRG4	34.90683	3.149471	-3.47033	4.18E-13
BUB1	8.335733	18.02637	1.112728	3.18E-22
FOLR1	448.5498	204.596	-1.13249	9.64E-11
TM4SF5	4.998029	15.4202	1.62539	0.022763
SLC5A9	2.301278	1.088279	-1.08039	1.04E-09
MACROD2	9.746607	3.868912	-1.33297	1.18E-09

DLEC1	3.698797	1.498455	-1.30358	1.14E-11
ANXA13	0.441931	2.681786	2.601302	0.002791
MMP10	7.421345	24.37647	1.715737	0.002025
MIR34C	3.603008	1.457867	-1.30534	2.88E-06
AL021068.1	6.791314	2.832441	-1.26165	0.003857
THBS4	23.0245	10.62241	-1.11606	9.77E-10
CAPSL	8.649137	4.259327	-1.02193	0.000166
GREB1L	0.939555	2.128337	1.179677	9.19E-09
STC2	6.901857	13.96339	1.016593	1.88E-07
CA4	5.06724	1.598374	-1.6646	5.92E-08
SCGB3A2	1951.12	427.2166	-2.19126	2.22E-16
C15orf48	89.92472	184.8363	1.039459	3.04E-11
MIR3671	4.780331	2.25655	-1.08299	2.41E-09
AC104971.3	1.499237	0.69236	-1.11464	2.08E-10
IL11	1.442437	2.912702	1.01385	1.05E-07
WFDC12	19.69248	0.585601	-5.07158	2.51E-09
FMO2	23.60541	10.85311	-1.12101	1.79E-19
CTSG	4.850927	1.903933	-1.34928	3.69E-11
Z83843.1	7.51216	3.755723	-1.00014	1.44E-06
SCGB3A1	2557.132	769.504	-1.73253	3.00E-14
KIAA0319	2.160721	5.061114	1.227942	0.030399
MTFR2	3.326541	6.960417	1.065151	1.24E-21
SCARNA5	85.13579	9.544475	-3.15703	0.049513
SNORD116-24	2.79338	1.37989	-1.01746	0.000299
STRA6	4.496364	9.209055	1.034294	1.66E-06
RSPH4A	7.249027	3.36722	-1.10623	3.74E-05
RASGRP2	5.656198	2.699945	-1.0669	9.34E-16
COL7A1	4.650821	11.77914	1.340677	1.53E-09
CENPA	6.000288	14.89877	1.31209	1.08E-26
CDCA5	11.2016	27.24757	1.282423	6.87E-27
ARC	1.949004	0.863029	-1.17526	9.24E-09
GFRA1	3.233558	0.836257	-1.9511	6.51E-25
KCNS1	1.884153	0.828778	-1.18486	0.007851
AMPD1	2.183238	0.879658	-1.31146	1.16E-14
LRRC66	0.719756	1.710371	1.248729	0.003266
KIF4A	8.143422	20.5444	1.335038	1.02E-25
IL1R2	3.15886	8.28692	1.391432	2.05E-10
FDCSP	42.49761	13.82022	-1.6206	3.04E-08
CXCL5	12.73213	39.0568	1.6171	0.001438
LY6G6C	0.643231	1.558752	1.276983	0.013631
SLC22A3	46.33869	20.79375	-1.15607	7.73E-16
FAM107A	10.5804	4.220254	-1.32599	1.55E-16
DKK1	9.44023	39.10557	2.05048	2.62E-07
INSL4	3.607552	11.75722	1.704455	0.006694

AC087284.1	1.550259	0.699997	-1.14709	0.041452
MIR30C2	1.959062	0.848922	-1.20646	2.16E-07
TMEM171	2.08031	4.630449	1.154354	1.16E-09
POLQ	1.470513	2.976885	1.017484	1.14E-16
ASPG	3.008547	1.438357	-1.06465	0.000148
LRRC71	2.408686	1.007231	-1.25785	3.79E-06
C1orf189	6.317447	2.932604	-1.10716	6.09E-06
GPR35	3.868679	7.978854	1.044341	3.20E-05
APOBEC1	1.273664	4.506521	1.82303	7.40E-05
AC058791.1	2.556921	0.739112	-1.79054	0.004598
CD19	8.19762	3.824387	-1.09998	4.48E-08
NDC80	7.02227	15.04186	1.098974	6.41E-24
GTSE1	5.068777	11.8272	1.222399	5.97E-26
MIR6071	4.890858	2.384859	-1.03618	2.06E-09
NCAPG	5.990511	14.15094	1.240147	5.38E-24
COL6A6	4.159034	1.483324	-1.48741	1.82E-18
EFNA2	0.814426	2.106989	1.371328	2.75E-05
ATP13A4	29.63738	13.87364	-1.09507	3.06E-11
AURKB	11.60939	28.489	1.295113	2.30E-26
AC012651.1	2.75744	1.204486	-1.19491	3.10E-12
CKAP2L	4.238616	9.198066	1.117737	6.04E-23
STAP1	4.599645	2.29762	-1.00138	8.50E-14
LRRN3	2.657863	1.105656	-1.26536	9.66E-20
MAP3K19	1.556502	0.66554	-1.22571	3.39E-06
GJB4	0.612412	1.950086	1.670962	0.00057
FAM83D	12.60798	26.16224	1.053149	3.19E-20
PTPRH	6.92544	16.91022	1.287918	8.87E-15
DNAI1	3.556103	1.528767	-1.21793	0.000173
AC015722.2	2.490173	0.958831	-1.3769	0.000227
ABCC2	3.021011	18.72988	2.632239	0.003015
ESPL1	3.367961	7.881438	1.226583	2.69E-22
KLK6	5.500987	24.20301	2.137424	1.59E-06
BCL2L1-AS1	2.647571	0.97663	-1.43878	9.31E-05
PGM5P4	1.761511	0.773066	-1.18815	2.17E-14
S100A2	51.77986	136.8976	1.402634	0.004284
FAM216B	9.195493	3.377172	-1.44511	9.69E-10
NTSR1	0.305978	3.580902	3.548821	0.008779
IL5RA	1.544857	0.629318	-1.29561	1.21E-13
ZBBX	2.463385	1.062744	-1.21285	8.12E-06
ELOVL3	1.53711	3.430208	1.158075	0.049192
AL138499.1	1.999478	0.940553	-1.08804	0.044406
FHL5	4.895574	2.16202	-1.1791	7.87E-18
ANKRD44-AS1	8.329505	3.489564	-1.25518	4.51E-11
SPAG5	12.2543	25.73343	1.070356	4.40E-23

GPR18	3.070492	1.512887	-1.02117	2.41E-08
MS4A2	4.936059	2.144312	-1.20284	1.76E-13
IGLV5-48	7.574431	3.264757	-1.21416	1.19E-05
CNIH3-AS2	0.912006	1.913054	1.068763	0.029947
MAGEA3	9.16992	27.04681	1.560477	7.94E-06
AL513282.1	4.731685	2.094711	-1.1756	0.000532
LINC01833	0.901789	1.880923	1.060579	8.84E-07
IRX1	6.087175	2.674642	-1.18643	1.28E-10
SP8	1.389935	2.903688	1.062869	1.67E-06
IL20RB	7.063219	16.4367	1.218523	0.000242
PLEKHG6	7.084132	14.25583	1.008889	3.16E-17
AC067747.1	1.765013	0.66425	-1.40988	5.87E-12
TMEM190	13.91251	6.867461	-1.01853	0.003997
C11orf97	3.32745	1.404493	-1.24437	0.000362
INHA	12.09432	26.25624	1.118331	0.006821
INA	2.211207	5.730598	1.373852	0.000612
HOPX	221.5441	88.39565	-1.32555	4.24E-13
AL645608.3	0.50077	1.54433	1.62476	0.030232
RAD51	4.731708	9.797041	1.049985	9.32E-24
HMGA1	209.2417	527.5828	1.334227	4.42E-28
KHDC1L	0.666902	3.159983	2.244369	0.00025
IGKV1D-16	89.76309	21.07151	-2.09083	8.99E-06
DNAI2	3.757248	1.417771	-1.40605	1.84E-07
AC124947.2	1.027433	2.115352	1.041854	2.98E-05
SEC14L6	9.191245	4.334795	-1.0843	5.45E-10
CCL19	138.0797	68.35503	-1.01438	3.69E-09
SYBU	19.83282	9.595188	-1.04751	1.25E-05
CASC15	1.58416	3.344878	1.078235	0.012706
AC004386.7	3.978933	1.122876	-1.82518	0.017982
PLA2G4F	11.3902	5.08515	-1.16343	2.52E-14
STX1A	10.01212	20.29665	1.019494	6.58E-16
LRRK2	36.09215	14.90763	-1.27563	5.09E-17
KLF15	13.27184	5.847137	-1.18257	1.01E-16
AC079467.1	6.201592	2.135398	-1.53813	3.83E-12
ZNF385B	15.92181	4.474094	-1.83134	7.94E-15
LINC00460	3.811908	11.11602	1.544054	0.008181
MYEOV	8.353801	25.22542	1.594373	1.70E-11
SFTPB	10023.09	4414.772	-1.18292	2.15E-15
C11orf88	3.050264	1.285605	-1.24649	5.25E-06
ANLN	13.51855	37.54167	1.473552	1.64E-27
IGKV2-26	4.769236	1.750051	-1.44636	2.29E-07
AQP7	4.633278	2.315525	-1.00069	9.22E-09
TCN1	26.57086	84.78464	1.673958	0.000133
GCLC	25.02763	61.33093	1.293093	1.52E-07

CCNA2	15.03843	32.37974	1.106438	2.72E-23
NNAT	28.74679	4.530809	-2.66556	0.004136
PTCHD4	1.857482	0.876254	-1.08393	2.54E-10
RIC3	2.07471	0.841488	-1.30189	1.25E-17
PIMREG	4.864509	10.53031	1.114181	2.89E-25
FAM72B	0.689169	1.626546	1.238882	1.95E-21
RNU6-247P	5.077192	1.80911	-1.48875	7.05E-05
PCSK2	72.71552	12.86856	-2.49841	1.09E-09
RNU1-38P	1.937669	0.427768	-2.17942	1.05E-12
TCTE1	1.599449	0.677196	-1.23993	0.000312
OMG	4.560437	2.108911	-1.11267	7.24E-12
C5orf49	10.54425	5.176001	-1.02655	1.05E-09
PCDH7	5.044954	10.32613	1.033387	1.96E-05
EXO1	4.408636	10.70555	1.279955	2.18E-24
VWA3B	2.431903	1.187013	-1.03475	7.23E-05
AC103563.1	16.306	7.945543	-1.03718	0.000303
LYPD3	9.610043	38.18704	1.990468	3.03E-14
AC009093.3	2.302519	0.975824	-1.23852	1.93E-10
BTBD16	0.772087	1.992252	1.367565	0.014059
KCNJ8	25.31688	12.61429	-1.00504	1.52E-06
CEP55	13.49616	32.1095	1.250451	1.84E-26
TRAJ3	2.582756	1.250966	-1.04587	0.000134
CDHR3	8.327333	4.100875	-1.02192	8.07E-09
C6	3.556897	1.658739	-1.10053	1.21E-10
AC002563.1	3.10904	1.198206	-1.37559	3.40E-11
CDC45	7.269618	17.10243	1.23425	5.82E-25
PTGFR	3.357103	1.507035	-1.1555	4.53E-07
ARHGEF2-AS1	3.545883	1.554184	-1.18999	4.24E-09
SLC5A5	2.096818	6.878094	1.713807	0.023878
STEAP1B	1.571113	3.310429	1.075232	0.000412
FHOD3	2.064397	4.717143	1.192193	0.000474
ABCA8	4.338758	1.282303	-1.75854	5.50E-25
PRAC2	0.572888	1.553326	1.439035	0.001634
FABP4	17.5606	7.649511	-1.1989	1.29E-08
CD1B	3.911625	1.857832	-1.07415	1.70E-11
AC013275.1	3.557875	1.597166	-1.1555	4.37E-11
MAGEA6	7.394468	20.38867	1.463249	4.72E-05
GAPDH	1512.412	3088.372	1.029995	6.24E-36
GJB3	6.472019	25.79795	1.994969	7.77E-13
TFAP2A	3.98398	9.255316	1.216072	1.27E-13
WFDC5	21.27598	0.716345	-4.89243	0.000264
SUSD2	178.3392	66.82972	-1.41606	3.80E-16
LINC00942	4.96799	18.14826	1.869097	1.09E-05
SCN7A	9.978635	3.871964	-1.36578	6.20E-22

SCGB2A1	30.02677	11.76052	-1.3523	0.000539
RHPN2	16.84951	33.80533	1.004544	3.81E-16
S100A7	3.142628	36.6529	3.543884	2.18E-05
ALDH3B2	8.644416	17.95923	1.054885	2.06E-09
AZU1	2.579978	0.83855	-1.62139	3.53E-06
GPIHBP1	9.003329	3.454299	-1.38207	6.56E-11
MMRN1	7.361767	3.513557	-1.06712	1.09E-14
GTF2IP7	0.668772	1.776353	1.409332	1.71E-14
UHRF1	6.367517	13.65051	1.100152	2.30E-26
MIR4539	7.599384	3.585417	-1.08374	0.000104
LINC01518	0.912574	1.933737	1.083378	5.13E-05
C7	92.36984	26.97494	-1.7758	1.72E-24
CYP2B7P	201.6554	77.09991	-1.38709	4.67E-19
RPL13AP17	2.61042	0.764761	-1.7712	3.07E-15
CLGN	4.00066	10.28324	1.361985	0.004467
KLK8	3.284944	7.181008	1.128318	0.003145
MS4A1	15.17753	5.07532	-1.58037	1.62E-12
CACNA2D2	51.40856	17.15066	-1.58374	6.48E-21
TNS4	9.683906	38.13978	1.977636	4.27E-09
ELANE	2.526867	1.025809	-1.30059	1.71E-09
IGKV6D-21	26.90028	13.35546	-1.01019	2.84E-05
AL132989.2	1.5334	0.717436	-1.09581	0.001881
AC079949.2	0.843162	2.499101	1.567527	0.004209
PLK1	10.79692	24.56853	1.186191	5.41E-27
RGS22	1.45336	0.678194	-1.09962	5.53E-12
HLA-DRB5	1163.306	565.8174	-1.03982	5.38E-13
LINC00941	0.54758	3.005574	2.456499	7.14E-07
C1orf61	0.554039	1.35783	1.293244	0.000615
ANGPT1	7.292495	3.547506	-1.03961	1.06E-15
CLSPN	3.13623	6.394618	1.027827	1.21E-16
RRM2	20.88585	49.07925	1.232587	1.05E-26
SBSPON	4.51246	1.998715	-1.17484	1.56E-09
NPR1	12.23589	6.096184	-1.00514	4.63E-11
ECT2	20.03594	41.9233	1.065162	5.34E-21
LPL	59.36259	27.2577	-1.12289	5.26E-13
SLPI	3320.351	1136.441	-1.54681	7.09E-05
MIR548V	3.573166	0.881379	-2.01937	0.000275
AC005256.1	0.903559	2.15698	1.255322	0.000596
SLC6A4	2.328926	0.75342	-1.62814	3.44E-10
CDC25A	2.653962	5.951825	1.165184	1.62E-25
MECOM-AS1	2.012927	0.720641	-1.48194	8.43E-09
CPA3	60.74258	29.9068	-1.02223	1.05E-10
DNAAF1	3.83722	1.496054	-1.3589	7.68E-09
ANKRD36BP2	3.681108	1.758762	-1.06558	9.28E-07

KRT6B	4.40418	9.348216	1.085817	1.06E-07
PLPPR4	2.928026	1.428553	-1.03537	1.22E-12
SYNGR4	0.78586	1.705615	1.117948	3.80E-09
RNF186	1.383367	3.838316	1.47229	0.012104
AC010735.2	2.029601	4.063082	1.001378	0.000833
SIGLEC17P	2.317627	1.158528	-1.00036	1.29E-12
AL133466.1	3.091944	1.357833	-1.18721	2.70E-12
CD109	8.272416	17.85832	1.110216	1.57E-08
FCRL1	2.396542	0.793728	-1.59424	7.95E-15
SNORA74B	13.95959	1.580247	-3.14303	0.001008
MIR7152	1.563262	0.730983	-1.09665	0.000217
IGLV8-61	297.5654	147.21	-1.01533	2.72E-05
CFLAR-AS1	1.986482	0.95822	-1.05179	0.000167
CCL14	2.100492	0.7306	-1.52357	8.08E-21
AL138760.1	1.510911	3.091754	1.033007	0.005422
SDCBP2	18.45532	39.07646	1.082263	2.15E-05
FAM92B	8.525472	3.533793	-1.27056	6.97E-09
IGHV1-45	37.0724	16.98088	-1.12643	1.10E-06
KCP	0.931266	2.562473	1.460272	1.19E-13
CLC	3.329111	1.635605	-1.02531	0.008416
RNU1-67P	2.69565	1.23713	-1.12364	0.034781
AC007878.1	2.755275	0.956385	-1.52653	0.000756
ACTL8	0.703859	2.160547	1.618038	0.000342
OGN	12.11652	3.208397	-1.91705	3.46E-13
TRAJ18	1.72017	0.770802	-1.15812	5.06E-06
VWA3A	3.226235	1.155865	-1.48088	8.73E-09
HSD17B6	54.31985	15.38189	-1.82025	9.05E-17
DCDC2B	2.263449	0.956988	-1.24195	1.14E-07
PLAU	131.8054	269.8908	1.033967	1.09E-08
AKR1C7P	0.837668	1.753082	1.065444	0.033882
IGLV3-13	3.086371	1.435865	-1.10399	4.05E-07
KRT20	5.886419	56.89398	3.272814	0.006449
DLGAP5	8.635376	21.16025	1.293026	2.86E-24
RN7SKP51	4.409141	2.116633	-1.05873	2.40E-11
AKAP14	2.825817	1.263757	-1.16095	1.64E-06
PVALB	2.604741	0.968371	-1.42751	1.12E-08
LINC02657	1.367436	3.487041	1.35053	4.66E-12
NPW	6.06538	13.71	1.176559	3.10E-05
CFAP221	7.562697	3.733609	-1.01833	7.21E-15
CTSV	2.717283	10.11785	1.896666	2.30E-26
ERVMER34-1	3.092294	6.349629	1.037995	1.54E-05
AC112777.1	0.967611	1.969908	1.02563	2.07E-17
C8orf34-AS1	16.44589	7.637198	-1.10661	4.92E-13
CD79B	17.39882	8.608109	-1.01522	7.57E-11

CASQ2	1.675261	0.713343	-1.23172	5.80E-17
MAGEC1	0.987806	3.134571	1.665969	0.000276
ARL14	3.112236	13.34928	2.100738	8.15E-06
FCRL3	2.980965	1.362084	-1.12996	4.94E-08
AC079384.1	6.020577	2.805232	-1.10178	0.000162
LANCL1-AS1	1.588105	0.579712	-1.4539	1.01E-12
AC061975.6	0.782165	1.597241	1.030037	0.000428
LINC00973	2.081595	7.850736	1.915138	0.002523
ARNTL2	8.817929	22.74132	1.366804	3.27E-15
CASP14	0.338668	3.429273	3.33996	5.77E-05
SFTPA2	6236.173	2422.303	-1.36428	2.62E-12
ITGA8	10.25252	5.01901	-1.0305	2.02E-18
SAPCD2	9.13326	18.8512	1.045454	5.75E-24
AFF3	2.918417	1.2248	-1.25264	4.17E-18
AC007686.1	0.54675	1.611031	1.55903	0.046774
GNG4	3.26055	10.53363	1.691816	1.05E-07
VGF	1.865943	12.52622	2.746974	1.06E-08
LINC01843	1.904374	4.721051	1.309791	2.15E-06
CENPF	9.667578	20.299	1.070182	3.24E-19
RNU6-341P	3.192721	1.193135	-1.42003	0.00251
BCHE	4.701629	2.218952	-1.08328	1.75E-08
CD22	7.640213	3.331577	-1.19741	2.64E-13
IGHD6-25	8.48835	3.77182	-1.17022	0.000421
NXPH4	5.102134	12.2441	1.262914	0.000548
SCARNA7	34.79962	5.152097	-2.75584	0.013457
BTLA	2.247892	1.10445	-1.02524	3.25E-14
PEBP4	97.85481	21.35947	-2.19577	6.47E-16
MFAP4	199.7363	85.95596	-1.21643	2.31E-17
LINC02577	1.732561	4.325026	1.319803	3.21E-10
COL6A5	2.534687	1.024911	-1.30631	2.11E-15
AC016831.4	2.038439	0.39613	-2.36342	0.001612
AGER	192.5813	56.07609	-1.78001	1.17E-10
GCNT3	16.78493	35.14947	1.066336	0.007471
VEGFD	12.55879	3.277373	-1.93809	5.49E-20
BLK	3.690211	1.445201	-1.35243	1.09E-11
RPS29P11	38.48462	18.3434	-1.06902	2.25E-11
IGLV1-41	33.54742	14.71518	-1.1889	0.000124
TCL1A	3.005503	1.449068	-1.05248	3.96E-06
SNRPGP4	2.507013	0.93648	-1.42065	0.000153
MS4A15	46.85656	23.14831	-1.01734	7.55E-08
KYNU	3.639531	10.94937	1.589023	0.010631
DLC1	34.2527	15.75763	-1.12017	3.92E-20
DNASE1L3	4.025935	1.504369	-1.42016	1.14E-14
CDK5R2	1.391096	3.006223	1.11173	2.14E-07

IGKV3OR2-5	1.886506	0.657554	-1.52054	1.52E-06
AL353662.1	2.230084	0.483289	-2.20614	0.008077
F2	0.174587	4.320315	4.62912	4.53E-08
UPK1B	7.676359	28.76747	1.905944	0.014008
LINC02313	0.767503	2.314545	1.592485	7.85E-05
ABCA6	2.041797	0.999385	-1.03073	2.50E-19
EPS8L3	2.988817	9.396566	1.652559	0.000416
CYP2B6	2.399033	0.993093	-1.27245	0.016053
IGHJ3P	72.55002	31.51723	-1.20284	1.17E-06
PKMYT1	4.980827	10.3048	1.048859	4.90E-26
ADH1B	40.878	9.286787	-2.13807	3.38E-27
CYP2F1	1.902998	0.731988	-1.37838	1.15E-05
LINC01629	0.53669	1.8016	1.747119	0.024624
MAGEC2	3.508536	8.724139	1.314144	3.69E-05
AC090181.3	2.82352	1.005224	-1.48998	0.000253
AP005262.1	0.623253	1.828242	1.552568	0.004672
FKBP9P1	1.661677	3.706772	1.157523	0.000357
CPB2	21.69587	4.336153	-2.32293	6.34E-09
LHFPL3-AS2	13.39634	5.103531	-1.39227	2.05E-15
IGLV7-43	131.5506	65.37405	-1.00883	3.31E-07
SULT2B1	9.166691	19.53749	1.091772	1.00E-09
IGHV3-22	4.522247	1.911141	-1.24261	3.45E-06
AC005077.4	1.691246	6.010815	1.829474	1.77E-22
AC114760.2	1.780972	0.692503	-1.36277	1.54E-06
MCM10	3.45869	7.756421	1.165165	5.37E-21
MIR4537	7.018049	3.261708	-1.10544	3.29E-06
SLC15A1	1.516171	4.666468	1.621899	9.83E-05
FOSL1	10.41274	33.457	1.683959	1.71E-13
TIMM9P2	1.849411	0.576429	-1.68185	0.030872
SNORA80B	7.712322	2.157832	-1.83758	0.002082
RHOF	2.414089	4.972344	1.042447	1.80E-10
SMIM24	2.643667	7.581899	1.520019	4.08E-05
ORC1	4.591303	10.62481	1.210462	4.80E-24
AC108215.1	4.858397	1.29413	-1.9085	7.03E-14
MIR553	2.864116	1.325388	-1.11168	0.018335
SYT13	5.850894	15.39997	1.396198	0.027161
AL353804.2	1.653289	0.793127	-1.05972	0.004134
ANXA8	1.110327	2.634292	1.246431	0.04746
AC012213.4	0.87165	2.464163	1.499276	3.56E-08
CTSH	443.8114	204.4459	-1.11823	5.81E-14
CLEC17A	1.526672	0.690307	-1.14508	1.04E-10
IGHV3-16	1.710171	0.648645	-1.39864	2.61E-06
TRIM31	4.582804	14.27234	1.638919	0.002738
JCHAIN	1574.499	730.44	-1.10806	1.12E-15

KRT6A	49.21032	149.8072	1.606074	1.66E-08
KRT83	0.729742	1.775345	1.282641	0.000293
CFAP73	6.301577	3.017281	-1.06246	0.000205
FOXD1	0.88305	2.970325	1.750053	0.001975
GPX3	305.1449	147.0045	-1.05363	2.67E-13
IGF2BP1	1.041387	5.690791	2.450123	4.09E-09
AC011298.1	0.447398	1.859446	2.055241	0.004641
AC115989.1	1.988744	0.682416	-1.54314	1.17E-08
CCDC60	1.580013	0.738063	-1.09812	6.73E-08
RN7SL8P	5.888074	1.99409	-1.56206	1.27E-12
UCA1	3.545838	13.84773	1.965451	0.001079
AC027288.3	3.140825	1.000236	-1.6508	1.51E-11
LINC02086	0.587549	1.330399	1.179079	0.00887
KRT81	13.3187	66.06621	2.31046	5.97E-06
SFTPC	1914.133	327.0315	-2.54919	8.50E-14
PBK	9.745494	19.59659	1.007796	1.55E-17
BUB1B	6.719478	15.71611	1.225823	1.65E-25
AC010789.1	1.430613	3.904323	1.448439	4.41E-06
GALNT14	9.65814	19.56613	1.018541	1.44E-08
MIR421	3.173761	1.225618	-1.37268	2.40E-06
CDC6	8.229485	19.80915	1.267293	7.81E-26
CADM3	4.288019	1.899102	-1.17499	9.97E-12
DUOXA1	18.56609	7.835841	-1.24451	4.77E-12
IGHV3-42	3.433947	1.591968	-1.10906	7.45E-06
DNAH9	2.043105	0.724313	-1.49608	7.17E-08
AL033397.2	1.856036	4.249282	1.194994	0.005307
ALOX15	11.17049	4.624836	-1.27222	2.23E-05
AC007684.1	2.494632	0.990901	-1.33202	0.018365
MORN5	6.777501	3.126217	-1.11634	7.30E-05
CYP17A1	2.50699	0.363588	-2.78558	1.34E-13
LINC01781	3.271276	1.250035	-1.38788	9.26E-12
AC016813.1	3.755547	0.425264	-3.14259	6.87E-11
HBQ1	0.483902	1.677353	1.793399	0.003464
ACKR1	29.06001	12.24753	-1.24654	4.29E-16
ATP1A2	1.800464	0.677584	-1.4099	1.19E-18
AC092071.1	11.16652	4.754096	-1.23194	2.38E-15
LY6D	14.013	28.19096	1.008467	0.005984
ERICH3	2.688992	1.095112	-1.29599	1.53E-05
AC092868.2	2.923858	10.10564	1.789216	0.042052
INSYN1	1.683708	0.766916	-1.1345	8.06E-16
ITGB1-DT	0.46532	2.701732	2.537589	1.34E-12
PRR15	10.60518	23.76189	1.16388	6.95E-07
CASC8	0.722115	1.730805	1.261143	2.31E-08
ATP13A4-AS1	4.391858	0.874079	-2.329	9.58E-11

PAX8	2.059601	4.857613	1.237883	0.042643
ARMC2-AS1	1.786108	0.687044	-1.37835	0.000337
HMGA2	0.989216	6.280692	2.666567	2.20E-10
RANBP20P	2.023705	0.194702	-3.37766	0.003116
AL136452.1	3.264339	1.514762	-1.1077	1.35E-11
NCAPH	8.37077	19.74018	1.237703	3.22E-25
CD40LG	4.867081	2.185291	-1.15523	5.67E-20
UBE2S	14.72247	30.51866	1.051671	2.53E-25
SERPINB5	4.374333	21.26788	2.281541	2.11E-08
AL096712.1	1.575583	0.633767	-1.31386	0.017194
TINAG	1.003716	2.443538	1.283621	0.001571
SLIT3	10.78528	4.642902	-1.21596	4.62E-14
LBP	7.401451	18.94909	1.356248	0.00111
ADGRF4	2.991954	8.742043	1.546882	2.75E-15
BTNL9	5.995401	2.042065	-1.55383	2.41E-18
KREMEN2	1.114303	2.471001	1.148954	2.94E-15
CDHR2	0.96337	4.52852	2.232877	3.19E-06
AP003119.3	1.454181	2.995023	1.04236	1.09E-07
KRT75	1.110217	2.8226	1.346183	0.016032
HSD17B13	6.018543	2.077505	-1.53456	7.15E-15
AL513548.1	1.740173	0.510289	-1.76984	2.67E-05
REG4	5.859474	93.167	3.990976	0.000615
SCUBE1	1.509245	0.723506	-1.06075	8.51E-08
SLCO4A1	5.994444	12.49108	1.0592	1.26E-06
KIF15	3.345342	6.922264	1.04909	3.50E-19
F11	2.152501	0.582898	-1.8847	5.17E-14
LHFPL3	2.475909	1.083736	-1.19194	2.06E-13
SNORD89	9.799351	4.764209	-1.04045	0.03175
PTGDS	131.8706	55.85793	-1.23929	1.19E-20
TRGJP2	5.568629	2.393163	-1.2184	4.93E-06
TRAJ4	1.587471	0.658118	-1.27031	4.26E-06
C20orf85	45.73293	19.32169	-1.24301	6.91E-06
AL109914.1	2.741052	1.18787	-1.20635	2.54E-06
TICRR	1.359064	3.159613	1.217134	4.47E-23
SNTN	4.714569	2.110014	-1.15987	2.67E-10
DMBT1	118.3427	55.09919	-1.10287	2.16E-06
SPDYC	1.043969	3.686494	1.820171	0.00063
FAM83A	34.94668	106.446	1.606894	2.11E-17
KRT19	677.6211	1379.245	1.025328	8.78E-17
PTCSC3	12.51849	4.838927	-1.3713	4.69E-15
IGKV1-33	14.45569	6.941819	-1.05825	0.000118
SCGB1A1	1429.472	378.217	-1.9182	3.85E-08
CNMD	67.3991	8.825194	-2.93303	3.56E-06
LRRC18	1.707881	0.693777	-1.29966	2.75E-08

SELENBP1	263.7155	122.1129	-1.11077	1.07E-17
AC113349.1	1.678911	0.510386	-1.71786	1.28E-07
MDFI	21.11861	43.76027	1.051107	1.37E-13
IL23A	5.028868	10.22996	1.024495	9.71E-06
CD1E	8.634801	4.165104	-1.05181	2.61E-14
GNMT	3.369482	1.637619	-1.04093	1.08E-15
UBE2SP1	1.273212	2.657205	1.061437	7.87E-15
CRISP2	2.355633	1.116861	-1.07666	0.000244
NEK2	9.948446	22.29321	1.164061	5.04E-24
AC020978.3	2.332244	1.112165	-1.06835	1.68E-10
TFF1	158.446	418.8674	1.402503	0.000494
CCNB1	33.36363	69.05826	1.049538	8.02E-24
BIRC5	20.25631	47.91212	1.242019	1.75E-25
PRR11	8.296261	19.75385	1.251601	1.08E-26
SGO1	2.442726	5.351688	1.131502	2.28E-23
PI3	23.0905	130.927	2.503392	0.000489
AF127577.3	0.866306	1.848885	1.093707	0.018309
FCRLA	6.389945	2.727922	-1.228	3.12E-10
PKIB	8.903348	18.86151	1.083025	5.59E-05
AL445493.3	2.837463	1.015374	-1.48259	2.83E-10
FAM171A2	3.641083	7.480229	1.038715	1.30E-05
PSMD10P1	2.317778	0.862549	-1.42606	0.000138
MYO3B	0.795125	1.807156	1.184468	0.007214
BCL2L10	1.285264	2.594944	1.013638	1.26E-12
CA3	5.793391	2.60544	-1.15288	1.81E-18
CAPN3	2.357066	0.994569	-1.24485	1.07E-13
KIF2C	12.43709	29.48267	1.245219	1.05E-26
AC027288.1	3.986161	1.475835	-1.43347	9.49E-11
FAM83F	1.177629	3.099396	1.396102	5.52E-06
OIP5	4.273373	9.556788	1.16115	4.42E-22
CFAP77	3.304822	1.451965	-1.18657	2.61E-06
IGKV1OR2-6	19.73712	8.019903	-1.29925	1.47E-06
LINC02471	5.506104	2.001361	-1.46005	3.00E-08
C4BPA	451.6563	225.339	-1.00313	1.07E-10
MUC2	0.1847	1.886215	3.352237	6.28E-06
PDX1	1.027794	2.64954	1.366191	0.009549
CDHR4	5.056094	2.142206	-1.23893	0.000284
MIR29B2CHG	3.253342	1.330679	-1.28976	9.69E-14
PRAP1	2.562851	8.693425	1.762175	0.004847
AC073149.1	2.345478	0.881122	-1.41247	4.25E-11
AKR1B10	53.44454	228.2888	2.094746	0.004467
MIR374B	2.165531	0.794501	-1.4466	6.84E-08
AC007849.1	2.053733	0.467732	-2.13449	3.43E-05
SFTPD	839.5427	252.406	-1.73386	9.99E-16

UGT2B7	0.83024	2.597854	1.64572	0.00265
NUS1P2	0.778494	1.73615	1.157134	0.003015
AL135999.3	23.52224	11.12739	-1.07991	5.39E-11
LINC02323	0.583152	1.869999	1.681095	8.35E-14
AP005233.2	4.20354	8.929676	1.087003	4.08E-10
FCER1A	24.36783	11.90272	-1.03369	4.76E-13
HJURP	8.082666	18.81599	1.219056	2.82E-26
FA2H	16.90235	36.0465	1.092635	1.51E-08
CHIA	18.76661	3.674288	-2.35263	4.10E-14
LINC00861	2.497357	1.209492	-1.046	3.25E-14
TMEM132D	1.992016	0.794259	-1.32655	3.09E-10
SMOX	10.00744	21.25013	1.086398	3.72E-15
C1QTNF7	3.289119	1.256725	-1.38803	5.65E-21
ACOXL	4.10206	2.040238	-1.00761	2.10E-16
SKA3	4.697849	10.31442	1.13459	1.08E-26
CYCSP6	1.547054	4.887017	1.65943	6.87E-06
SPC25	5.723549	11.52751	1.010098	1.07E-23
SBSN	1.682794	4.630018	1.460159	1.81E-09
RAD51AP1	7.867304	17.1381	1.123266	2.35E-21
ADAMTS7P3	2.021608	0.378418	-2.41745	2.93E-14
AL133304.2	3.151001	0.900415	-1.80715	2.13E-08
GDF10	7.058116	2.434256	-1.5358	1.38E-14
MIR31HG	0.797829	2.386577	1.580791	1.82E-05
FAM30A	2.806819	1.282727	-1.12972	2.91E-09
INMT	42.49677	12.58689	-1.75543	6.13E-26
RN7SKP80	4.73913	2.164498	-1.13059	2.09E-05
RNY4P25	1.562712	0.632091	-1.30585	0.001697
ZIC2	1.127504	2.552314	1.178674	0.000151
FAM83A-AS1	4.817085	12.82583	1.412819	9.44E-15
CDCA3	3.67124	9.146698	1.316984	1.23E-28
CDC20	32.05225	78.53096	1.292836	5.61E-27
SPATA18	11.42283	5.396363	-1.08186	1.35E-11
PENK	5.401546	1.923216	-1.48985	6.62E-13
RSPO1	1.796916	0.373276	-2.26721	6.87E-18
AC112722.1	1.838605	0.804507	-1.19243	3.78E-13
IGHVIII-67-2	3.717997	1.439517	-1.36894	0.000102
C1orf87	1.821356	0.707105	-1.36502	9.15E-08
GJB5	3.461796	8.973945	1.374222	0.001464
ABI3BP	12.38963	5.329633	-1.21703	7.07E-21
GCKR	0.6524	1.520668	1.220876	0.001098
PKP2	4.843353	13.08353	1.433674	3.13E-10
DUOX1	24.40845	9.120077	-1.42026	1.93E-12
AL590226.1	2.306076	0.992151	-1.21681	1.10E-16
PWAR5	1.997299	0.805131	-1.31076	6.81E-05

WIF1	104.2882	13.11947	-2.9908	6.00E-15
RPL32P1	2.798415	1.281367	-1.12693	2.47E-13
AC006270.1	2.485301	1.2083	-1.04044	0.02044
HOXA10	1.763621	4.451047	1.335605	5.91E-05
LY6K	5.401648	12.67429	1.230433	4.35E-07
IGKV2D-30	7.628288	3.392169	-1.16915	0.000196
SLC46A2	12.29564	4.473104	-1.4588	1.44E-12
AUNIP	2.225686	5.232659	1.233294	1.35E-24
TUBAL3	0.81745	1.850231	1.178502	2.74E-05
AC141273.2	2.823299	1.302062	-1.11658	1.86E-06
TESMIN	1.571716	4.420508	1.491872	5.78E-18
IGF2BP3	2.781786	7.65619	1.460616	1.72E-11
GAS6-AS1	3.85396	1.816653	-1.08506	2.43E-11
TPX2	29.66551	73.95455	1.317852	1.64E-26
LINC02351	2.832997	1.198853	-1.24067	1.91E-07
AL157931.1	0.676045	1.511715	1.160996	0.000402
TUBA4B	4.862668	2.246025	-1.11437	6.88E-05
SOX14	4.462477	1.411138	-1.66099	0.000491
NR4A3	10.36488	4.882988	-1.08587	2.51E-06
UBE2C	55.68021	135.9267	1.287593	1.86E-23
ALKAL2	1.937587	0.606299	-1.67616	8.65E-17
SLCO4A1-AS1	1.519107	3.808106	1.32585	0.000119
SRGAP3-AS2	7.120019	2.910336	-1.2907	1.30E-06
AC084375.1	10.24172	4.120093	-1.31371	1.39E-09
CIDEC	0.263306	2.230019	3.082245	6.70E-05
PSMD10P2	0.593743	1.754643	1.563267	2.81E-15
RNU6-1010P	3.013917	1.379633	-1.12736	0.010558
AC010998.3	2.537102	1.043352	-1.28196	3.44E-12
SNORA47	3.910924	0.841245	-2.21691	0.003354
C8orf34	1.420363	0.683158	-1.05597	5.89E-13
WDR38	9.755802	4.41326	-1.14442	0.0011
PITX1	7.886131	19.87179	1.333332	1.67E-12
TNXB	8.188685	3.015864	-1.44106	1.09E-11
AL161431.1	1.252581	5.003725	1.998099	0.033557
DEPDC1	3.712578	8.406569	1.179096	2.06E-22
AC120498.2	4.688292	1.954411	-1.26233	7.20E-06
PLA2G1B	37.54757	10.63232	-1.82026	5.85E-15
RTBDN	0.727301	1.51253	1.056339	9.36E-05
RNU6ATAC39P	1.640396	0.804639	-1.02763	0.026442
GJB2	29.42703	68.93861	1.22817	2.42E-11
EMCN	10.84049	5.312076	-1.02908	1.10E-18
NWD1	3.318206	1.387772	-1.25763	5.83E-05
AC027288.2	5.432964	2.353043	-1.20721	7.12E-08
GPR87	11.61172	27.96544	1.268063	2.56E-07

RAD54L	4.341181	8.724251	1.006944	1.10E-20
HHATL	3.516812	1.042408	-1.75435	2.05E-05
LINC01116	2.303507	5.479521	1.250218	0.000121
CHRDL1	25.90077	9.793252	-1.40314	9.26E-25
TTLL10	1.849673	0.74429	-1.31333	3.98E-05
HES2	1.648139	3.594351	1.124894	0.008065
ECT2L	2.059156	0.881488	-1.22404	2.16E-07
LGI3	6.504469	1.343596	-2.27533	8.70E-13
F5	6.253764	12.59212	1.009725	0.004069
ELFN1-AS1	1.525728	3.661015	1.262746	0.000123
LINC02555	4.529917	1.296628	-1.80472	3.47E-09
SELENOOLP	0.735347	4.307903	2.550489	0.000787
MIR6124	2.392805	1.155819	-1.04979	0.02324
BNIP3P40	1.694183	0.546388	-1.63259	0.00208
ADAMTS8	5.909452	1.861119	-1.66685	4.44E-20
DYNLRB2	4.188629	1.853655	-1.1761	1.01E-09
CFAP52	4.148708	1.685501	-1.29948	2.01E-05
AC053527.1	1.697287	0.630047	-1.4297	0.00171
MKI67	11.7011	28.07015	1.262393	1.09E-23
AC023421.1	20.50828	6.004021	-1.77221	1.56E-10
C16orf89	610.6091	182.0099	-1.74623	7.46E-20
AC026347.1	7.061783	2.652529	-1.41266	4.02E-05
AC022784.1	2.031806	7.715429	1.924984	1.05E-07
EIF4EBP1	81.76891	179.1927	1.131888	7.58E-22
CDCA8	17.18691	35.09331	1.029886	1.28E-25
KIF11	11.37947	24.40529	1.10076	7.55E-24
GLYATL2	5.077882	2.160855	-1.23262	0.036416
TSPAN19	1.994755	0.791072	-1.33433	0.000157
KIFC1	16.47371	34.47574	1.065416	1.86E-25
LINC01612	5.184096	2.157328	-1.26485	2.26E-09
CYP4Z2P	3.172137	0.593491	-2.41816	7.36E-20
PGC	4251.697	1071.91	-1.98785	1.10E-17
GJB6	3.73349	9.032147	1.274544	0.015588
GDA	0.685673	2.055319	1.58377	0.0014
OIT3	2.371857	1.132556	-1.06644	3.19E-09
NR4A1AS	6.753403	2.564182	-1.39712	9.01E-11
PRR20G	0.626355	2.817125	2.169172	1.04E-05
IL22RA1	2.782815	6.842513	1.297981	1.13E-07
PNCK	0.74659	1.721133	1.204971	3.91E-05
FTX	2.568706	1.11792	-1.20022	2.23E-13
NAPSA	1963.769	936.2423	-1.06867	6.10E-16
CYP2A6	15.65559	1.232675	-3.66681	2.49E-12
CABCOCO1	6.293333	2.771992	-1.1829	1.04E-09
AC094019.2	1.680435	0.78818	-1.09224	9.73E-08

CST5	2.787536	1.277797	-1.12533	1.37E-10
TMEM100	13.59707	5.560624	-1.28998	7.10E-12
RNASE1	1760.007	806.3158	-1.12616	1.73E-17
AC018629.1	1.395728	4.700321	1.751741	3.71E-05
CENPE	2.813607	5.868108	1.060475	1.82E-19
HCN2	0.929105	2.082052	1.164092	3.07E-06
STOML3	3.512336	1.608706	-1.12653	2.72E-05
LINC01108	1.686178	0.520881	-1.69473	0.000116
HOXA9	0.343184	1.733479	2.336618	0.021869
ANGPTL4	46.57877	93.96277	1.012417	2.19E-09
SLC16A12	3.813724	1.807345	-1.07733	1.49E-05
ENTPD2	3.416166	6.999567	1.034888	1.31E-06
E2F7	1.530202	3.285841	1.10254	2.26E-16
TPSD1	7.490365	3.620313	-1.04892	0.002102
DTHD1	2.418088	0.958882	-1.33444	4.75E-09
MALAT1	366.4345	116.6045	-1.65193	5.50E-05
SOX15	2.842533	7.803946	1.457027	9.71E-06
FENDRR	3.104469	1.214536	-1.35394	5.27E-11
DAAM2	17.65799	8.80031	-1.0047	2.10E-19
AL357093.2	5.549662	2.366756	-1.22949	1.12E-08
PADI3	2.251743	7.097982	1.656367	0.00021
ATP5MC1P4	1.325031	2.841704	1.10073	2.02E-17
AC099850.4	11.98176	27.54461	1.20093	9.63E-23
LINC02163	0.918213	2.067372	1.170897	0.001807
SLC2A1	56.00997	175.6852	1.649237	1.70E-26
SFTPA1	5175.67	1950.477	-1.40792	2.16E-13
FOXM1	12.53955	34.6769	1.467489	1.47E-27
PACRG	2.292913	1.131556	-1.01887	2.36E-06
ACADL	3.81198	1.512717	-1.3334	1.00E-12
MGP	362.3864	180.5586	-1.00506	4.70E-19
HTR1D	2.021742	5.434234	1.426478	7.18E-11
AC133963.1	4.253596	1.782744	-1.25458	2.46E-11
DEFB1	26.35596	67.07411	1.347627	0.002011
DNER	4.031041	8.362641	1.052806	0.029139
PLEKHH1	1.677311	3.641956	1.118563	5.40E-08
UCN2	0.467656	1.638717	1.809049	5.32E-13
MAMDC2	13.40013	4.88614	-1.45548	3.81E-22
ECRG4	6.808017	2.21642	-1.619	1.48E-16
LOXL2	21.29563	46.28281	1.119919	4.13E-13
SERPINB3	5.652848	20.01034	1.823696	0.011926
RNU4ATAC	6.367048	1.85484	-1.77933	0.000889
AC079210.1	4.006868	1.826372	-1.13349	6.11E-10
SLC16A1	9.880455	23.12857	1.227026	1.00E-06
SERPINB7	1.18444	2.739749	1.209839	0.000149

LAMP3	125.9324	61.93325	-1.02386	4.55E-12
FAM83B	1.466463	3.336058	1.185804	0.001346
CR2	12.27057	5.435716	-1.17466	1.75E-07
ZNF695	0.661107	1.419334	1.102258	1.41E-08
IGKV1D-8	30.06496	14.39014	-1.063	3.80E-06
AC068587.2	5.34372	2.440818	-1.13048	1.45E-11
CDH3	40.37802	86.08774	1.092238	1.87E-10
HILPDA	27.98081	58.24939	1.057805	4.12E-12
RNU6-1016P	10.10295	4.956624	-1.02735	0.003179
S100A9	1123.01	2419.115	1.107108	0.002455
IGLV3-16	22.46537	10.08328	-1.15574	5.57E-08
RAB3B	0.990183	3.659387	1.885834	3.49E-13
SERPINB4	1.675159	4.945488	1.561815	4.22E-05
AC116407.1	6.067072	2.897662	-1.06611	8.19E-12
KIF14	2.802096	6.207481	1.147501	2.95E-22
MAGEA12	4.830863	13.30588	1.461711	0.005476
SSXP10	2.185805	0.380235	-2.5232	4.60E-05
AC008268.1	26.79318	10.20205	-1.39301	2.82E-08
LAMC2	88.27186	215.2817	1.2862	1.27E-12
CDT1	10.7377	22.1511	1.044694	6.20E-25
FHL1	27.69868	11.4361	-1.27622	4.98E-19
AL162511.1	18.07795	5.004872	-1.85283	3.34E-17
AL513008.1	2.200413	0.845087	-1.3806	1.49E-05
CYP4B1	165.18	41.57968	-1.99009	6.67E-20
PRSS3	2.298637	11.62125	2.337915	1.97E-05
NXF3	1.690914	0.734821	-1.20234	0.000308
PLOD2	33.34029	67.35338	1.014483	8.97E-12
LRRC36	5.187012	1.483272	-1.80612	3.29E-16
MIR6774	1.810678	0.785174	-1.20545	3.21E-07
FAM166B	7.987641	3.648466	-1.13048	0.000106
CYP24A1	36.98194	118.405	1.678838	1.43E-07
LRRK2-DT	15.66138	6.141302	-1.3506	1.63E-19
AC026785.3	3.67881	7.641789	1.054671	0.003379
TRIP13	12.74584	26.47355	1.054525	1.25E-20
AC090001.1	1.996807	0.822338	-1.27989	0.000258
PPP2R2C	2.320097	6.212932	1.421089	0.002868
MYOZ1	6.021326	2.926699	-1.04081	5.78E-11
IGKV3D-7	4.318645	1.86314	-1.21284	1.26E-05
CFAP100	2.541304	1.002651	-1.34175	8.70E-08
ABCA3	151.7635	69.77407	-1.12106	8.07E-15
FURIN	170.3013	363.4485	1.093661	0.000213
AC005076.1	2.361963	0.496348	-2.25056	2.50E-06
HPDL	2.592027	5.478757	1.079768	5.38E-11
C22orf15	2.224847	0.935788	-1.24945	4.06E-06

AC005722.2	2.372514	0.993903	-1.25524	7.65E-06
AL049555.1	2.428728	6.044555	1.315435	0.000372
GKN2	51.45243	10.43282	-2.30211	2.73E-10
A2ML1	0.711255	4.084373	2.521675	0.029791
MLXP1	1.081558	3.870386	1.839367	1.17E-05
AC108451.2	0.777674	3.882828	2.319871	0.020624
ST8SIA6-AS1	1.161672	3.379322	1.540531	0.049022
LNCAROD	0.251546	1.805005	2.843107	3.56E-06
NFE4	2.824621	8.399497	1.572246	2.46E-05
RHOV	21.5628	93.17061	2.111331	2.24E-20
IGHV1-24	531.7257	247.7798	-1.10162	0.003974
IGKV1OR2-108	63.6555	29.1227	-1.12814	3.31E-06
IGLV1-36	167.4023	55.27641	-1.59858	3.69E-07
NEIL3	3.266827	7.131404	1.126296	1.22E-14
AURKA	17.40841	35.20343	1.015932	9.73E-20
RXRG	2.366306	1.178037	-1.00625	9.91E-10
CFAP161	1.458677	0.679888	-1.10129	2.53E-06
AL139041.1	3.372377	1.41628	-1.25166	2.29E-16
ARHGEF4	1.950195	4.429152	1.183412	1.09E-07
GGTLC1	68.195	24.07578	-1.50208	1.22E-15
LINC00922	4.333482	1.928899	-1.16775	0.007904
C6orf118	2.904622	1.178231	-1.30173	7.15E-06
MIR1972-1	1.462739	0.706184	-1.05056	0.0024
MBL1P	4.15241	1.841131	-1.17336	7.55E-16
IGHV1OR16-3	4.826992	2.066228	-1.22412	7.37E-06
HSPA6	12.86938	29.05412	1.1748	0.00827
AP001011.1	2.947323	1.219326	-1.27332	3.52E-05
SLC26A9	50.58383	21.16503	-1.25699	8.04E-09
KCNF1	0.973229	2.880567	1.565502	0.00093
BTG2	255.636	121.2546	-1.07605	2.68E-24
SAXO2	4.122966	1.941829	-1.08627	1.58E-07
FGF19	1.134439	5.697938	2.328462	0.000983
MELTF	5.329884	14.52996	1.446855	9.51E-14
PTGES	47.00674	101.2999	1.107694	3.06E-06
LINC00337	0.694486	1.824404	1.393409	9.95E-17
AGTR2	14.25955	3.783653	-1.91408	2.72E-06
SFRP5	3.729819	1.650143	-1.17651	0.013374
FSCN1	65.23657	136.7187	1.067458	2.90E-10
GUCY2C	1.357873	3.773393	1.474514	0.005616
CNGA4	1.898943	0.940371	-1.01389	4.08E-06
LPAL2	2.402671	1.108162	-1.11647	1.55E-16
CHEK1	5.309168	11.47776	1.112284	4.42E-26
SLC7A5	61.53102	125.1215	1.023943	4.08E-13
MELK	10.40898	22.7779	1.129806	1.24E-21

DSG3	1.19759	3.014855	1.331955	0.013173
KRT17	62.65465	242.3194	1.951417	7.44E-05
LINC01559	0.600134	3.599053	2.584261	1.98E-05
KIF23	5.850359	13.29332	1.184105	1.18E-26
NCCRP1	6.846524	18.98493	1.471411	0.046094

Supplementary Table S7. GO enrichment analysis of differentially expressed genes between high and low programmed cell death score group

Category	ID	Description	Counts	qvalue
BP	GO:0140014	mitotic nuclear division	40	2.62E-12
BP	GO:0000070	mitotic sister chromatid segregation	30	2.62E-12
BP	GO:0007059	chromosome segregation	41	4.12E-11
BP	GO:0000819	sister chromatid segregation	31	4.86E-11
BP	GO:0051983	regulation of chromosome segregation	22	8.36E-10
BP	GO:0000280	nuclear division	44	1.24E-09
BP	GO:0098813	nuclear chromosome segregation	34	1.74E-09
BP	GO:0048285	organelle fission	46	2.52E-09
BP	GO:0030071	regulation of mitotic metaphase/anaphase transition	16	2.71E-09
BP	GO:0007091	metaphase/anaphase transition of mitotic cell cycle	16	4.02E-09
BP	GO:1902099	regulation of metaphase/anaphase transition of cell cycle	16	4.02E-09
BP	GO:0044784	metaphase/anaphase transition of cell cycle	16	6.52E-09
BP	GO:0010965	regulation of mitotic sister chromatid separation	16	7.93E-09
BP	GO:0051306	mitotic sister chromatid separation	16	1.63E-08
BP	GO:1905818	regulation of chromosome separation	16	2.52E-08
BP	GO:0006959	humoral immune response	38	2.81E-08
BP	GO:0007088	regulation of mitotic nuclear division	24	3.89E-08
BP	GO:0019730	antimicrobial humoral response	22	3.89E-08
BP	GO:0033047	regulation of mitotic sister chromatid segregation	16	8.07E-08
BP	GO:0051304	chromosome separation	18	8.33E-08
BP	GO:1902850	microtubule cytoskeleton organization involved in mitosis	22	8.96E-08
BP	GO:0033045	regulation of sister chromatid segregation	17	9.38E-08
BP	GO:0007094	mitotic spindle assembly checkpoint	11	4.34E-07
BP	GO:0031577	spindle checkpoint	11	4.34E-07
BP	GO:0071173	spindle assembly checkpoint	11	4.34E-07
BP	GO:0071174	mitotic spindle checkpoint	11	4.34E-07
BP	GO:0007052	mitotic spindle organization	19	4.34E-07
BP	GO:0051783	regulation of nuclear division	24	4.61E-07
BP	GO:0033046	negative regulation of sister chromatid segregation	12	6.83E-07

BP	GO:0045841	negative regulation of mitotic metaphase/anaphase transition	11	7.66E-07
BP	GO:0051985	negative regulation of chromosome segregation	12	8.47E-07
BP	GO:1902100	negative regulation of metaphase/anaphase transition of cell cycle	11	9.93E-07
BP	GO:2000816	negative regulation of mitotic sister chromatid separation	11	1.79E-06
BP	GO:1905819	negative regulation of chromosome separation	11	2.33E-06
BP	GO:0051310	metaphase plate congression	13	3.35E-06
BP	GO:0033048	negative regulation of mitotic sister chromatid segregation	11	3.85E-06
BP	GO:0007093	mitotic cell cycle checkpoint	21	3.94E-06
BP	GO:0045839	negative regulation of mitotic nuclear division	12	4.08E-06
BP	GO:0008608	attachment of spindle microtubules to kinetochore	10	4.40E-06
BP	GO:0007051	spindle organization	22	4.40E-06
BP	GO:0050000	chromosome localization	14	9.75E-06
BP	GO:0051303	establishment of chromosome localization	14	9.75E-06
BP	GO:1901987	regulation of cell cycle phase transition	38	1.13E-05
BP	GO:0019731	antibacterial humoral response	12	1.57E-05
BP	GO:0051784	negative regulation of nuclear division	12	1.87E-05
BP	GO:0000075	cell cycle checkpoint	23	2.31E-05
BP	GO:1901990	regulation of mitotic cell cycle phase transition	35	3.35E-05
BP	GO:0090307	mitotic spindle assembly	12	4.39E-05
BP	GO:1901988	negative regulation of cell cycle phase transition	25	7.23E-05
BP	GO:0007080	mitotic metaphase plate congression	10	9.53E-05
BP	GO:0010948	negative regulation of cell cycle process	29	0.000158
BP	GO:1901991	negative regulation of mitotic cell cycle phase transition	23	0.000221
BP	GO:0007018	microtubule-based movement	29	0.000249
BP	GO:0070942	neutrophil mediated cytotoxicity	5	0.000419
BP	GO:0003341	cilium movement	17	0.00042
BP	GO:0000076	DNA replication checkpoint	6	0.000512
BP	GO:0070268	cornification	14	0.000612
BP	GO:0051225	spindle assembly	14	0.000738
BP	GO:2001251	negative regulation of chromosome organization	16	0.00081
BP	GO:0033044	regulation of chromosome organization	27	0.000954
BP	GO:0042742	defense response to bacterium	26	0.001728

BP	GO:0051988	regulation of attachment of spindle microtubules to kinetochore	5	0.001728	
BP	GO:0045787	positive regulation of cell cycle	28	0.002258	
BP	GO:0051383	kinetochore organization	6	0.002371	
BP	GO:0045930	negative regulation of mitotic cell cycle	25	0.003167	
BP	GO:0001578	microtubule bundle formation	12	0.003483	
BP	GO:0051315	attachment of mitotic spindle microtubules to kinetochore	5	0.003546	
BP	GO:0000083	regulation of transcription involved in G1/S transition of mitotic cell cycle	7	0.004384	
BP	GO:0044843	cell cycle G1/S phase transition	23	0.004921	
BP	GO:0061844	antimicrobial humoral immune response mediated by antimicrobial peptide	10	0.005064	
BP	GO:0044839	cell cycle G2/M phase transition	21	0.005793	
BP	GO:0051873	killing by host of symbiont cells	6	0.00586	
BP	GO:0001909	leukocyte mediated cytotoxicity	12	0.006073	
BP	GO:0031570	DNA integrity checkpoint	15	0.006721	
BP	GO:0007568	aging		23	0.006941
BP	GO:0034698	response to gonadotropin	6	0.006955	
BP	GO:0033314	mitotic DNA replication checkpoint	4	0.007765	
BP	GO:0051231	spindle elongation	4	0.007765	
BP	GO:0006270	DNA replication initiation	7	0.007765	
BP	GO:0090068	positive regulation of cell cycle process	22	0.007813	
BP	GO:0051883	killing of cells in other organism involved in symbiotic interaction	6	0.008008	
BP	GO:0051384	response to glucocorticoid	14	0.008008	
BP	GO:0050829	defense response to Gram-negative bacterium	10	0.008218	
BP	GO:0001906	cell killing		15	0.012275
BP	GO:0002697	regulation of immune effector process	29	0.013149	
BP	GO:0007143	female meiotic nuclear division	6	0.013783	
BP	GO:0000086	G2/M transition of mitotic cell cycle	19	0.014171	
BP	GO:0000281	mitotic cytokinesis	9	0.014782	
BP	GO:0035404	histone-serine phosphorylation	4	0.015219	
BP	GO:0051255	spindle midzone assembly	4	0.015219	
BP	GO:0051656	establishment of organelle localization	27	0.01606	
BP	GO:1902749	regulation of cell cycle G2/M phase transition	17	0.01606	
BP	GO:0051984	positive regulation of chromosome segregation	6	0.018206	
BP	GO:0045861	negative regulation of proteolysis	24	0.018369	
BP	GO:0071459	protein localization to chromosome, centromeric region	5	0.018369	
BP	GO:0017144	drug metabolic process	7	0.018491	

BP	GO:0044774	mitotic DNA integrity checkpoint	11	0.018704
BP	GO:0097529	myeloid leukocyte migration	17	0.019418
BP	GO:0031341	regulation of cell killing	10	0.019418
BP	GO:0002440	production of molecular mediator of immune response	21	0.019418
BP	GO:0031960	response to corticosteroid	14	0.020095
BP	GO:0045132	meiotic chromosome segregation	10	0.020757
BP	GO:0000082	G1/S transition of mitotic cell cycle	20	0.020986
BP	GO:0007100	mitotic centrosome separation	4	0.024656
BP	GO:0050930	induction of positive chemotaxis	4	0.024656
BP	GO:0060707	trophoblast giant cell differentiation	4	0.024656
BP	GO:1904668	positive regulation of ubiquitin protein ligase activity	4	0.024656
BP	GO:0010466	negative regulation of peptidase activity	19	0.026496
BP	GO:0002377	immunoglobulin production	16	0.026866
BP	GO:0071715	icosanoid transport	7	0.026866
BP	GO:1901571	fatty acid derivative transport	7	0.026866
BP	GO:0030595	leukocyte chemotaxis	17	0.028194
BP	GO:0000910	cytokinesis	14	0.02881
BP	GO:0051321	meiotic cell cycle	18	0.029051
BP	GO:1903046	meiotic cell cycle process	15	0.030189
BP	GO:0051299	centrosome separation	4	0.030269
BP	GO:0002449	lymphocyte mediated immunity	23	0.03092
BP	GO:0031145	anaphase-promoting complex-dependent catabolic process	9	0.03153
BP	GO:0061640	cytoskeleton-dependent cytokinesis	10	0.03153
BP	GO:0060706	cell differentiation involved in embryonic placenta development	5	0.032738
BP	GO:0010951	negative regulation of endopeptidase activity	18	0.032738
BP	GO:0002460	adaptive immune response based on somatic recombination of immune receptors built from immunoglobulin superfamily domains	23	0.034276
BP	GO:0140013	meiotic nuclear division	14	0.034973
BP	GO:0006956	complement activation	14	0.036554
BP	GO:0002523	leukocyte migration involved in inflammatory response	4	0.036554
BP	GO:0016572	histone phosphorylation	6	0.038872
BP	GO:0010389	regulation of G2/M transition of mitotic cell cycle	15	0.039111
BP	GO:0035082	axoneme assembly	8	0.039111
BP	GO:0042445	hormone metabolic process	16	0.039111
BP	GO:0031099	regeneration	15	0.040242

BP	GO:0051382	kinetochore assembly	4	0.044519	
BP	GO:0045840	positive regulation of mitotic nuclear division	7	0.048875	
BP	GO:0019835	cytolysis		5	0.048875
BP	GO:0050855	regulation of B cell receptor signaling pathway	5	0.048875	
BP	GO:0071624	positive regulation of granulocyte chemotaxis	5	0.048875	
BP	GO:0032465	regulation of cytokinesis	9	0.048927	
BP	GO:1902750	negative regulation of cell cycle G2/M phase transition	10	0.049346	
CC	GO:0000779	condensed chromosome, centromeric region	21	4.05E-08	
CC	GO:0005819	spindle		36	1.24E-07
CC	GO:0000775	chromosome, centromeric region	25	1.57E-07	
CC	GO:0000940	condensed chromosome outer kinetochore	8	1.57E-07	
CC	GO:0000777	condensed chromosome kinetochore	18	2.17E-07	
CC	GO:0000793	condensed chromosome	26	2.58E-07	
CC	GO:0000776	kinetochore	20	3.17E-07	
CC	GO:0042599	lamellar body	8	5.87E-07	
CC	GO:0062023	collagen-containing extracellular matrix	35	4.63E-06	
CC	GO:0000780	condensed nuclear chromosome, centromeric region	9	1.11E-05	
CC	GO:0000922	spindle pole	19	2.21E-05	
CC	GO:0051233	spindle midzone	9	2.78E-05	
CC	GO:0005874	microtubule	33	3.42E-05	
CC	GO:0072686	mitotic spindle	16	7.35E-05	
CC	GO:0098687	chromosomal region	28	8.23E-05	
CC	GO:0005876	spindle microtubule	11	9.74E-05	
CC	GO:0000778	condensed nuclear chromosome kinetochore	6	0.000149	
CC	GO:0000794	condensed nuclear chromosome	13	0.000286	
CC	GO:0005875	microtubule associated complex	16	0.000534	
CC	GO:0019814	immunoglobulin complex	16	0.000688	
CC	GO:0030496	midbody		17	0.000717
CC	GO:0031225	anchored component of membrane	16	0.001117	
CC	GO:0005771	multivesicular body	9	0.001546	
CC	GO:0009897	external side of plasma membrane	27	0.002988	
CC	GO:0005922	connexin complex	5	0.004704	
CC	GO:0005930	axoneme		12	0.004704
CC	GO:0031514	motile cilium	16	0.005073	
CC	GO:0097014	ciliary plasm	12	0.005073	
CC	GO:1990023	mitotic spindle midzone	4	0.008066	
CC	GO:0005871	kinesin complex	7	0.009881	

CC	GO:0045120	pronucleus	4	0.013071	
CC	GO:0099568	cytoplasmic region	17	0.022226	
CC	GO:0032838	plasma membrane bounded cell projection cytoplasm	15	0.022226	
CC	GO:0031362	anchored component of external side of plasma membrane	4	0.023608	
CC	GO:0005921	gap junction	5	0.02624	
CC	GO:0042383	sarcolemma	11	0.027429	
CC	GO:0036157	outer dynein arm	3	0.036587	
CC	GO:0005680	anaphase-promoting complex	4	0.037088	
CC	GO:0014704	intercalated disc	6	0.039252	
CC	GO:0035371	microtubule plus-end	4	0.041639	
CC	GO:0031233	intrinsic component of external side of plasma membrane	4	0.047174	
CC	GO:0045171	intercellular bridge	7	0.047174	
CC	GO:0030018	Z disc		10	0.047174
MF	GO:0008017	microtubule binding	28	5.70E-06	
MF	GO:0015631	tubulin binding	31	7.33E-05	
MF	GO:0003777	microtubule motor activity	12	0.000636	
MF	GO:0004867	serine-type endopeptidase inhibitor activity	12	0.005646	
MF	GO:0003774	motor activity	14	0.005646	
MF	GO:0019865	immunoglobulin binding	6	0.006342	
MF	GO:0046906	tetrapyrrole binding	14	0.013936	
MF	GO:0005506	iron ion binding	14	0.013936	
MF	GO:0030414	peptidase inhibitor activity	16	0.013936	
MF	GO:0020037	heme binding	13	0.017294	
MF	GO:0005243	gap junction channel activity	5	0.018614	
MF	GO:0004866	endopeptidase inhibitor activity	15	0.023075	
MF	GO:0061134	peptidase regulator activity	17	0.025457	
MF	GO:0004252	serine-type endopeptidase activity	14	0.025457	
MF	GO:0008201	heparin binding	14	0.025457	
MF	GO:0061135	endopeptidase regulator activity	15	0.028572	
MF	GO:0005504	fatty acid binding	6	0.036967	
MF	GO:0035173	histone kinase activity	4	0.046468	
MF	GO:0004857	enzyme inhibitor activity	23	0.046468	

Supplementary Table S8. KEGG enrichment analysis of differentially expressed genes between high and low programmed cell death score group

Category	Description	Counts	qvalue
hsa04110	Cell cycle	15	0.001266
hsa00590	Arachidonic acid metabolism	9	0.009479
hsa04640	Hematopoietic cell lineage	11	0.014591
